# Collagen‐Binding IL‐15 Immunocytokine Armed Oncolytic Adenovirus Establishes a Self‐Reinforcing Immunostimulatory Milieu to Potentiate Antitumor Immunity

**DOI:** 10.1002/advs.77691

**Published:** 2026-09-16

**Authors:** Yaqi Yang, Xiaoxiao Liu, Junqiang Ding, Jinliang Xu, Jianhua Zhang, Hanchang Zhang, Rong Yang, Sha Li, Lu Zheng, Runping Su, Yue Zhao, Jianxiao Wu, Hao Li, Changyou Zhan, Xihui Gao

**Affiliations:** ^1^ Key Laboratory of Medical Molecular Virology of MOE/NHC/CAMS School of Basic Medical Sciences Department of Pharmacy Shanghai Institute of Infectious Disease and Biosecurity Shanghai Pudong Hospital Fudan University Shanghai China; ^2^ Department of Otolaryngology‐Head and Neck Surgery, Massachusetts Eye and Ear Infirmary Harvard Medical School Boston Massachusetts USA; ^3^ Department of Pharmacology School of Basic Medical Sciences Fudan University Shanghai China; ^4^ Shanghai Ninth People's Hospital Shanghai Jiao Tong University School of Medicine Shanghai China; ^5^ Organ Transplantation Clinical Medical Center of Xiamen University, Department of General Surgery, Xiang'an Hospital of Xiamen University School of Medicine Xiamen University Xiamen China

**Keywords:** collagen‐binding domain, interleukin ‐ 15, immunosuppressive tumor microenvironment, oncolytic adenovirus, triple‐negative breast cancer, tumor immunotherapy

## Abstract

The immunosuppressive microenvironment limits immunotherapy in triple‐negative breast cancer (TNBC). Interleukin‐15 (IL‐15) activates cytotoxic lymphocytes but is limited by rapid clearance and poor tumor retention. Here, we developed an oncolytic adenovirus encoding IL15C‐CBD, an immunocytokine comprising an IL‐15/IL‐15Rα sushi‐domain complex fused to a collagen‐binding domain, thereby establishing a self‐reinforcing immunostimulatory milieu within tumors. Intratumoral administered OV‐IL15C‐CBD lyses tumor cells and secretes IL15C‐CBD that anchors to extracellular matrix collagens, forming a localized cytokine reservoir. In the murine 4T1 TNBC model, this strategy profoundly remodeled the microenvironment by recruiting CD8^+^ T cells and natural killer cells, depleting regulatory T cells, and polarizing macrophages. toward an M1‐like phenotype. Consequently, OV‐IL15C‐CBD induced significant tumor regression and extended survival without evident systemic toxicity. This therapeutic advantage was further validated in the EMT6 breast tumor model, yielding consistent antitumor efficacy and prolonged survival. Collectively, anchoring immunocytokines to the tumor stroma via oncolytic viruses provides a potentially safe and effective localized immunotherapy for TNBC.

## Graphical abstract

Repurposing the dense tumor stroma from a physical barrier into an anchoring scaffold, an engineered oncolytic adenovirus encoding IL15C‐CBD overcomes the immunosuppressive microenvironment in triple‐negative breast cancer. Intratumoral viral replication secretes this collagen‐anchored immunocytokine, forming a localized cytokine depot. This spatial confinement drives robust lymphocyte infiltration, inducing profound tumor regression and prolonged survival without systemic toxicity.

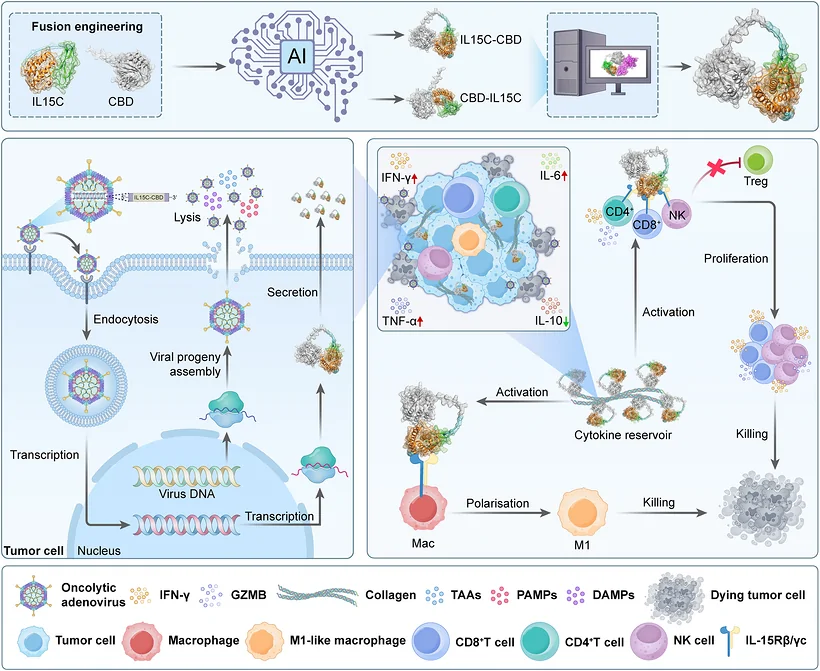

## 1 Introduction

Cancer immunotherapy has revolutionized the treatment of several malignancies; however, its efficacy remains limited in many solid tumors owing to immunosuppressive tumor microenvironments (TMEs) that restrict immune infiltration and effector function [1, 2, 3]. This challenge is exemplified by triple‐negative breast cancer (TNBC), defined by a dense extracellular matrix (ECM) and an immunosuppressive TME, both of which present formidable barriers to effective immune cell infiltration and durable antitumor immunity [4, 5, 6, 7]. Overcoming stromal exclusion and reprogramming the tumor immune milieu are thus critical unmet needs in the management of TNBC [8, 9].

Cytokine‐based immunotherapy holds great promise for enhancing antitumor immunity [10, 11]. Interleukin (IL)‐15 has emerged as a pivotal regulator of both innate and adaptive immune responses [12, 13]. Unlike other ILs, such as IL‐2, IL‐15 selectively activates CD8^+^ T cells and natural killer (NK) cells without expanding regulatory T cells (Tregs), making it a particularly attractive therapeutic candidate [14]. Furthermore, IL‐15/IL‐15Rα superagonist (e.g., N‐803) fusion proteins are engineered to mimic the physiological trans‐presentation of IL‐15 and exhibit increased stability and potency [15, 16, 17]. However, the clinical efficacy of these constructs remains constrained by their rapid clearance from circulation, poor intratumoral retention, and severe systemic toxicity due to off‐target distribution [18].

Harnessing the TME for localized therapy offers a compelling strategy to address these challenges [19]. The tumor ECM, dominated by type I and III collagen, not only facilitates tumor progression and immune evasion but also serves as a physical barrier that hinders the infiltration and persistence of effector immune cells [20]. Paradoxically, this collagen‐rich stroma presents a unique opportunity, as its abundant fibers provide a natural scaffold for anchoring therapeutic proteins selectively within the tumor [21]. Tethering cytokines to the collagen matrix could improve intratumoral retention and local efficacy while minimizing systemic exposure and toxicity [22]. Engineered proteins comprising collagen‐binding domain (CBD) fused to cytokines have been shown to exhibit prolonged intratumoral retention and enhanced local immune responses. However, these constructs remain suboptimal because systemically administered fusions risk off‐target accumulation in normal collagen‐rich tissues, such as the liver and kidneys, as well as at sites of tissue injury [23, 24]. Preclinical studies indicate that such off‐target deposition may not only elicit systemic toxicity but also reduce therapeutic efficacy by depleting cytokine availability at the tumor site [25].

Oncolytic viruses (OVs) offer an orthogonal yet complementary solution by providing tumor‐selective replication and transgene expression [26]. These viruses induce immunogenic cell death and trigger the release of damage‐associated molecular patterns (DAMPs), pathogen‐associated molecular patterns (PAMPs), and tumor‐associated antigens (TAAs), thereby priming immune responses within the TME [27]. Furthermore, their replicative nature enables the prolonged intratumoral expression of immunostimulatory payloads [28]. However, no strategy to date has surmounted these convergent challenges. It remains unclear how best to achieve robust, sustained infiltration of immune cells within the collagen‐dense, immunosuppressive microenvironments characteristic of TNBC while avoiding off‐target toxicity. Addressing this critical knowledge gap is the primary aim of the present study.

**SCHEME 1:**
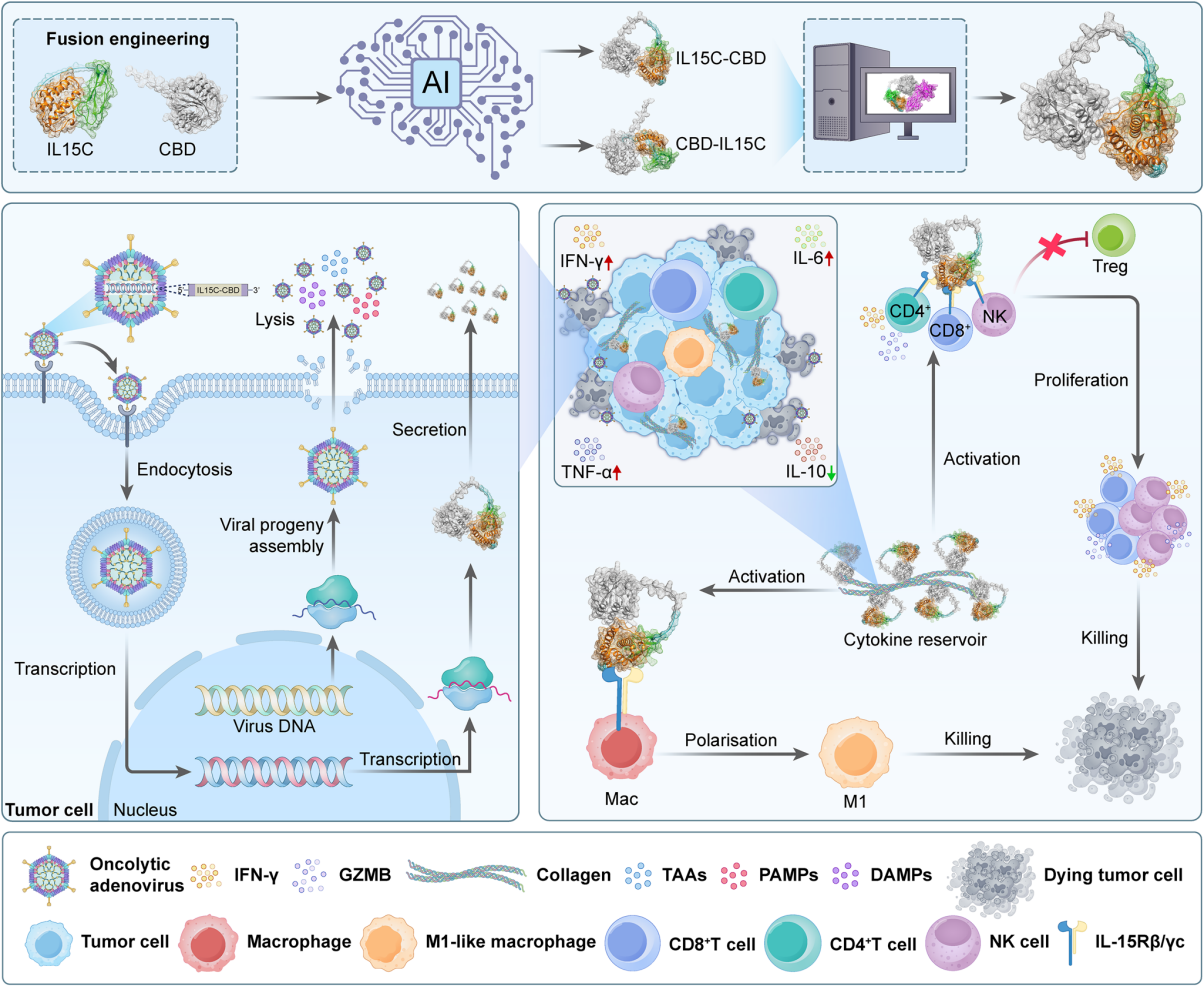
The engineered oncolytic adenovirus OV‐IL15C‐CBD remodels the tumor immune microenvironment by activating multiple immune pathways.

Here, we present a therapeutic platform that integrates oncolytic virotherapy with localized cytokine delivery to establish a self‐reinforcing immunostimulatory milieu within the TME, overcoming barriers to immune activation in TNBC (Scheme 1). Specifically, we engineered an oncolytic adenovirus (OV‐IL15C‐CBD) encoding a novel immunocytokine (IL15C‐CBD) in which the IL‐15 and IL‐15Rα sushi domain complex is fused to a high‐affinity CBD. Intratumoral administration of OV‐IL15C‐CBD drives selective tumor oncolysis and the secretion of IL15C‐CBD, which anchors to fibrillar collagens in the ECM. This spatial confinement creates a localized cytokine reservoir that sustains high levels of intratumoral IL‐15Rβ/γc signaling. Consequently, OV‐IL15C‐CBD induces robust infiltration and activation of NK cells and CD8^+^ T cells, markedly amplifying granzyme B (GZMB) and interferon (IFN)‐γ production. Crucially, these potent antitumor immune effects are achieved while minimizing systemic cytokine exposure and off‐target toxicity. By integrating selective oncolytic activity with tumor‐localized immune amplification, OV‐IL15C‐CBD transforms immune‐cold and treatment‐refractory tumors into immunologically active lesions. This platform provides a rational strategy for TME‐targeted immunotherapy and offers a clinically translatable approach for treating stroma‐rich malignancies.

Rational and computational protein engineering generated IL15C‐CBD, an immunocytokine integrating a superagonist comprising IL‐15 and IL‐15Rα with a CBD to enhance matrix affinity and stability. Encoding this construct within an oncolytic adenoviral vector restricts transgene expression to the tumor compartment. Following intratumoral delivery, OV‐IL15C‐CBD triggers immunogenic cell death and the release of TAAs, PAMPs, and DAMPs. Concurrently, secreted IL15C‐CBD anchors to stromal collagen fibers to establish a localized cytokine depot. This spatial confinement drives sustained engagement of IL‐15 receptor complexes on infiltrating lymphocytes, thereby expanding CD8^+^ T cell and NK cell populations while restricting regulatory T cell accumulation. The resulting immune cascade promotes M1 macrophage polarization and elevates proinflammatory cytokines such as IFN‐γ, tumor necrosis factor α (TNF‐α), and IL‐6, simultaneously suppressing immunosuppressive mediators like IL‐10. Ultimately, this dual mechanism of direct oncolysis and stroma‐anchored immunostimulation establishes a self‐reinforcing milieu for durable antitumor immunity.

## 2 Results

### 2.1 IL15C‐CBD Exhibits Dual Functionality in Collagen Binding and Immune Activation

**FIGURE 1:**
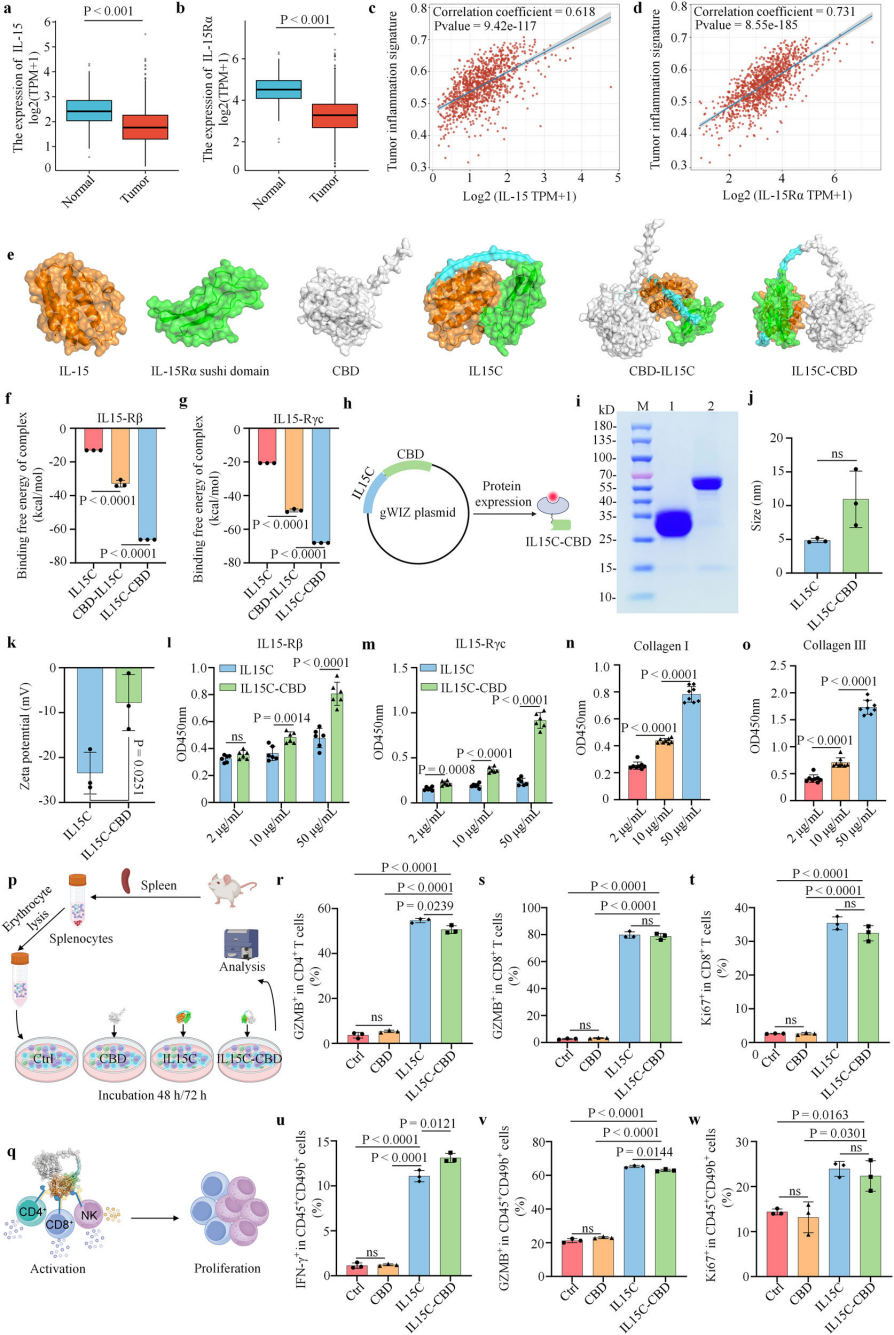
IL15C‐CBD mediates collagen anchoring and cytotoxic immune activation. Expression levels of IL‐15 (a) and IL‐15Rα (b) in BRCA and normal tissues, based on analysis of the TCGA and GTEx datasets. Spearman correlation of inflammatory pathway signatures with IL‐15 (c) and IL‐15Rα (d) expression in the BRCA dataset. (e) Structural models of IL‐15, IL‐15Rα sushi domain, CBD, IL15C, CBD‐IL15C, and IL15C‐CBD; peptide linkers are shown in blue. Binding free energies of IL15C, CBD‐IL15C, and IL15C‐CBD to IL‐15Rβ (f) and IL‐15Rγc (g) (n = 3/group). (h) Schematic representation of the gWIZ expression vector encoding IL15C‐CBD. (i) SDS‐PAGE analysis of purified IL15C and IL15C‐CBD (lane M, protein marker; lane 1, IL15C; lane 2, IL15C‐CBD). (j) Hydrodynamic diameter of IL15C and IL15C‐CBD measured by DLS (n = 3/group). (k) Zeta potential of IL15C and IL15C‐CBD (n = 3/group). ELISA analysis of the binding capacities of IL15C and IL15C‐CBD to IL‐15Rβ (l) and IL‐15Rγc (m) (n = 6/group). Dose‐dependent binding of IL15C‐CBD to type I (n) and type III (o) collagen was evaluated by ELISA (n = 8/group). (p) Schematic workflow for splenocyte isolation and in vitro stimulation. Splenocytes isolated from BALB/c mouse spleens following erythrocyte lysis were assigned to four groups (Ctrl, CBD, IL15C, IL15C‐CBD) and cultured for 48 h or 72 h before flow cytometric detection. (q) Schematic showing IL15C‐CBD activates CD4^+^ T cells, CD8^+^ T cells, and NK cells, and drives proliferation of CD8^+^ T cells and NK cells. Flow cytometric analysis of splenocytes treated with Ctrl, CBD, IL15C, or IL15C‐CBD, showing the frequencies of CD4^+^GZMB^+^ (r), CD8^+^GZMB^+^ (s), CD8^+^Ki67^+^ (t), CD45^+^CD49b^+^IFN‐γ^+^ (u), CD45^+^CD49b^+^GZMB^+^ (v), and CD45^+^CD49b^+^Ki67^+^ (w) cells (n = 3/group). The data are presented as the mean ± standard deviation (SD). Statistical significance was assessed using t‐tests and nonparametric tests (j, k), two‐way ANOVA with Tukey's multiple comparisons (l, m), and one‐way ANOVA with Tukey's multiple comparisons (f, g, n, o, r–w). ns indicates no significant difference. Image created with BioRender. com, with permission (h, p).

We first evaluated the therapeutic potential of IL‐15 signaling in breast cancer immunotherapy through bioinformatic analyses of pathway components across human malignancies. Transcriptomic analyses of The Cancer Genome Atlas (TCGA) and Genotype‐Tissue Expression (GTEx) datasets revealed a consistent pattern of IL‐15 mRNA downregulation in tumor tissues compared with adjacent normal tissues (Figure 1a). Higher IL‐15 expression showed a trend toward improved overall survival (OS, Figure S1a, *p* = 0.087) and disease‐free survival (DFS, Figure S1b, *p* = 0.11). Parallel analysis of IL‐15Rα revealed a nominally significant association with improved OS (*p* = 0.049, Figure S1c) and a marginal trend toward prolonged DFS (*p* = 0.071, Figure S1d), consistent with the reduced expression observed in tumor tissues (Figure 1b). Immune cell deconvolution analyses in BRCA revealed a strong association between IL‐15 and IL‐15Rα expression and tumor infiltration by cytotoxic CD8^+^ T cells, and immunostimulatory macrophages (Figure S2a,b). Notably, this relationship was also observed across other solid tumors, including lung adenocarcinoma, pancreatic adenocarcinoma, colorectal adenocarcinoma, and skin cutaneous melanoma. Pathway enrichment analyses substantiated these findings, revealing significant enrichment of tumor inflammation signatures (Figure 1c,d) and establishing IL‐15 signaling as a central regulator of antitumor immune responses.

Building upon prior efforts such as the N‐803 immunocytokine, which links IL‐15 to the sushi domain of IL‐15Rα to mimic natural trans‐presentation, we engineered an IL15C variant by fusing IL‐15 to the sushi domain of IL‐15Rα via a flexible (G_4_S)_3_ linker. We fused a CBD to either the N‐ or C‐terminus to increase tumor retention, producing CBD‐IL15C and IL15C‐CBD, respectively. We utilized AlphaFold2‐based structural modeling to assess whether the fusion configuration affects domain accessibility or introduces conformational clashes. The modeling confirmed the absence of significant steric hindrance in both configurations, with IL15C‐CBD displaying greater domain exposure (Figure 1e), a property anticipated to increase receptor accessibility and downstream signaling. The orientation of CBD fusion had a pronounced effect on receptor engagement. Both constructs retained high affinity for IL‐15Rβ and IL‐15Rγc; however, IL15C‐CBD exhibited the lowest binding free energy (Figure 1f,g; Figure S3a–f). This suggests that C‐terminal CBD fusion promotes protein stabilization and increases the accessibility of IL‐15 active sites, whereas N‐terminal fusion may partially obstruct receptor interaction.

We further evaluated the conformational consequences of CBD incorporation through hydrophobic cluster analysis using ProteinTools [29]. The results showed that IL15C maintains its tertiary structure through a single hydrophobic core (Cluster 0, Figure S4a). In contrast, CBD fusion gives rise to a dual‐domain hydrophobic architecture comprising six discrete clusters (Figure S4b). The hydrophobic surface area of the IL15C core expanded from 3008.84 to 3218.28 Å^2^ upon fusion, accompanied by an increase in mean intramolecular hydrophobic contacts from 2.92 to 3.04, indicating modest reinforcement of the IL15C fold (Tables S1 and S2). The CBD domain itself contributes two high‐density hydrophobic cores (Clusters 4 and 5, Figure S4b) with elevated mean contact numbers, imparting additional packing stability to the fusion protein. Consistent with these hydrophobic clustering results, hydrogen‐bond network analysis confirmed that the eight stabilizing networks of IL15C are largely retained in IL15C‐CBD, alongside extensive hydrogen‐bond networks intrinsic to CBD, collectively enhancing conformational rigidity (Figure S4c,d; Tables S3 and S4). Based on these structural and biophysical observations, we selected IL15C‐CBD for further functional evaluation.

Having established the structural rationale, we next characterized the biochemical and functional attributes of IL15C‐CBD. Following transient expression in human embryonic kidney (HEK)293F cells, both IL15C and IL15C‐CBD were purified to homogeneity. SDS‐PAGE and immunoblotting verified the expected molecular weights of 30 kDa and 56 kDa, respectively (Figure 1h,i; Figure S5). Dynamic light scattering (DLS) analysis revealed that, compared with IL15C, IL15C‐CBD displayed a slightly larger average particle size (10.93 nm versus 4.83 nm, Figure 1j) and a shift in zeta potential (‐7.78 mV versus ‐23.46 mV, Figure 1k). Eukaryotic expression of gWIZ‐CBD yielded a purified protein of approximately 26 kDa (Figure S6a,b) that bound type I collagen in a dose‐dependent manner (Figure S6c) yet showed no detectable interaction with IL‐15Rβ or IL‐15Rγc (Figures S6d,e, S7, and S8). Together, these data indicate that the CBD moiety retains collagen‐binding activity and does not directly engage IL‐15 receptor subunits, supporting its role as a collagen‐targeting module within the IL15C‐CBD fusion protein.

Quantitative binding analysis by ELISA demonstrated a substantially enhanced affinity of IL15C‐CBD for IL‐15Rβ (2.9‐fold increase relative to IL15C) and IL‐15Rγc (10.2‐fold increase) (Figure 1l,m). Moreover, only IL15C‐CBD exhibited dose‐dependent binding to type I and type III collagen (Figure 1n,o; Figures S9 and S10). We next determined whether this collagen‐binding activity translates to stromal retention within tumor tissue. We first characterized the ECM composition of 4T1 tumors by immunofluorescence staining for type I and type III collagen, using BALB/c mouse tail and skin as positive controls (Figure S11). Both subtypes were prominently distributed throughout the tumor stroma, confirming that this model recapitulates a collagen‐rich microenvironment. Ex vivo immunofluorescence analysis of 4T1 tumor cryosections incubated with purified IL15C‐CBD or IL15C further revealed extensive colocalization of IL15C‐CBD with stromal collagen, whereas IL15C showed negligible retention (Figure S12). This establishes that CBD fusion confers specific collagen‐tethering capacity within the native tumor matrix.

In vitro splenocyte activation assays revealed that both IL15C and IL15C‐CBD potently drove the cytotoxic functional differentiation of CD4^+^ T cells, CD8^+^ T cells, and NK cells, while concurrently promoting the proliferation of CD8^+^ T cells and NK cells (Figure 1p,q). Specifically, whereas CBD treatment alone produced no significant difference relative to untreated controls, both IL15C and IL15C‐CBD elicited comparable increases in the proportions of GZMB^+^ CD4^+^ T cells (50.61% for IL15C‐CBD and 54.68% for IL15C, Figure 1r; Figure S13a,b) and GZMB^+^ CD8^+^ T cells (78.67% for IL15C‐CBD and 79.78% for IL15C, Figure 1s; Figure S13c). They also promoted equivalent CD8^+^ T cell proliferation (32.41% for IL15C‐CBD and 35.37% for IL15C, Figure 1t; Figure S14a,b) and induced similar enhancements in NK cell IFN‐γ secretion (13.12% for IL15C‐CBD and 11.09% for IL15C, Figure 1u; Figure S13d), GZMB production (62.98% for IL15C‐CBD and 65.23% for IL15C, Figure 1v; Figure S13e), and proliferative expansion (22.35% for IL15C‐CBD and 23.93% for IL15C, Figure 1w; Figure S14c). The functional integrity of IL15C‐CBD was further maintained under collagen‐rich conditions, as co‐incubation with type I collagen, type III collagen, or both in combination left IL15C‐CBD‐mediated IFN‐γ and GZMB induction in CD8^+^ T cell and NK cell populations unaltered (Figure S15a–j). Separately, titration experiments using IL15C revealed a clear concentration‐dependent relationship, wherein stimulation at 10 µg/mL versus 2 µg/mL resulted in significantly greater IFN‐γ and GZMB secretion across CD4^+^ T cell, CD8^+^ T cell, and NK cell compartments alongside enhanced proliferative responses (Figures S16 and S17), providing a rational basis for dose optimization in subsequent in vivo studies. These findings establish IL15C‐CBD as a bifunctional immunocytokine that integrates collagen‐targeted tumor retention with potent immune activation unimpaired by the collagen‐rich TME.

### 2.2 Engineering of OV‐IL15C‐CBD and Assessment of Its Oncolytic Activity

**FIGURE 2:**
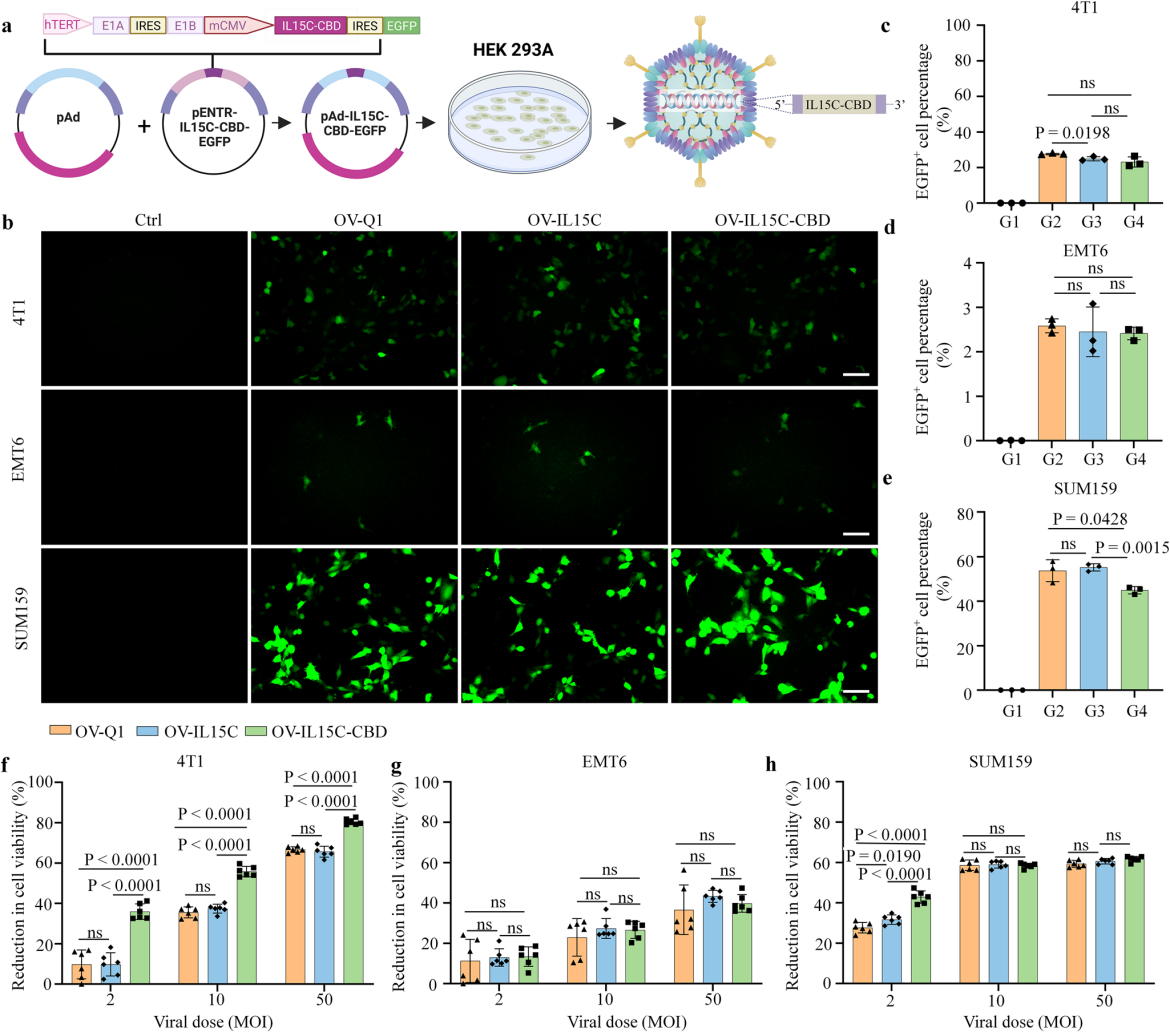
Construction and functional evaluation of engineered oncolytic adenoviruses. (a) Schematic of the OV‐IL15C‐CBD vector design, highlighting the IL15C‐CBD fusion gene insertion site. (b) Representative EGFP fluorescence images of 4T1, EMT6, and SUM159 cells under different treatment conditions (untreated control, OV‐Q1, OV‐IL15C, and OV‐IL15C‐CBD). Scale bar, 100 µm. Flow cytometric quantification of EGFP‐positive cells in 4T1 (c), EMT6 (d), and SUM159 (e) cells treated with different vectors (n = 3/group; G1, untreated control; G2, OV‐Q1; G3, OV‐IL15C; G4, OV‐IL15C‐CBD). Dose‐dependent oncolytic activity of OV‐Q1, OV‐IL15C, and OV‐IL15C‐CBD in 4T1 (f), EMT6 (g), and SUM159 (h) cells. Tumor cells were infected with the viruses at the indicated MOIs, and cell viability was assessed 48 h post‐infection (n = 6/group). The data are presented as the mean ± SD. Statistical significance was assessed using one‐way ANOVA with Tukey's multiple comparisons (c–e) or two‐way ANOVA with Tukey's multiple comparisons (f‐h). ns indicates no significant difference. Image created with BioRender. com, with permission (a).

Building upon the immunostimulatory properties of IL15C‐CBD, we engineered a novel oncolytic adenovirus using a human Ad5 backbone, with the human telomerase reverse transcriptase (hTERT) promoter inserted into the E1 region to confer tumor‐selective replication. The coding sequence for IL15C‐CBD was placed under the control of a murine cytomegalovirus (mCMV) promoter positioned downstream of the hTERT promoter to drive transgene expression, generating OV‐IL15C‐CBD (Figure 2a). For comparative studies, we constructed two control vectors, OV‐IL15C, which expresses IL15C alone, and OV‐Q1, a backbone vector lacking any immune effector genes (Figure S18a,b).

We evaluated the infectivity of the engineered viruses by transducing three breast cancer cell lines (4T1, EMT6, and SUM159) with each vector at a multiplicity of infection (MOI) of 1. EGFP fluorescence detected at 48 h post‐infection confirmed successful transduction across all three cell lines (Figure 2b). Flow cytometric analysis further demonstrated comparable levels of EGFP‐positive cells across OV‐Q1, OV‐IL15C, and OV‐IL15C‐CBD (Figure 2c–e), indicating that engineering the OVs to express the IL15C‐CBD immunocytokine did not affect viral infectivity. Viral genome replication was subsequently assessed by absolute quantification of E1A copy numbers at 48 h post‐infection across a panel of five cell lines (4T1, EMT6, SUM159, LLC, and A549) at an MOI of 10. All three viruses replicated their genomes efficiently in each cell line. In EMT6 (Figure S19b), SUM159 (Figure S19c), LLC (Figure S19d), and A549 (Figure S19e) cells, replication levels were broadly comparable among the three constructs. Notably, in 4T1 cells, both OV‐IL15C and OV‐IL15C‐CBD exhibited markedly elevated E1A copy numbers relative to OV‐Q1 (approximately 3.32‐fold and 2.98‐fold, respectively). In contrast, no significant difference was observed between OV‐IL15C and OV‐IL15C‐CBD (Figure S19a), indicating that IL15C‐CBD transgene incorporation does not significantly impair viral genome replication. Finally, we assessed the oncolytic potency of the engineered viruses at MOIs of 2, 10, and 50. All constructs demonstrated dose‐dependent cytotoxicity in 4T1 (Figure 2f), EMT6 (Figure 2g), and SUM159 (Figure 2h) breast cancer cells, as well as in LLC (Figure S20) and A549 (Figure S21) lung cancer cells. Notably, OV‐IL15C‐CBD induced a more pronounced reduction in 4T1 cell viability than OV‐Q1 and OV‐IL15C, suggesting enhanced cytotoxicity in this model. In the other tested cell lines, OV‐IL15C‐CBD showed broadly comparable oncolytic activity to OV‐Q1 and OV‐IL15C across a range of MOIs. Collectively, these findings demonstrate that incorporation of the IL15C‐CBD cassette preserves the infectivity, replicative capacity, and intrinsic lytic activity of the oncolytic adenoviral vector.

### 2.3 IL15C‐CBD Immunocytokine Secreted by OV‐IL15C‐CBD Promotes Immune Cell Activation and Potentiates Cytotoxicity

**FIGURE 3:**
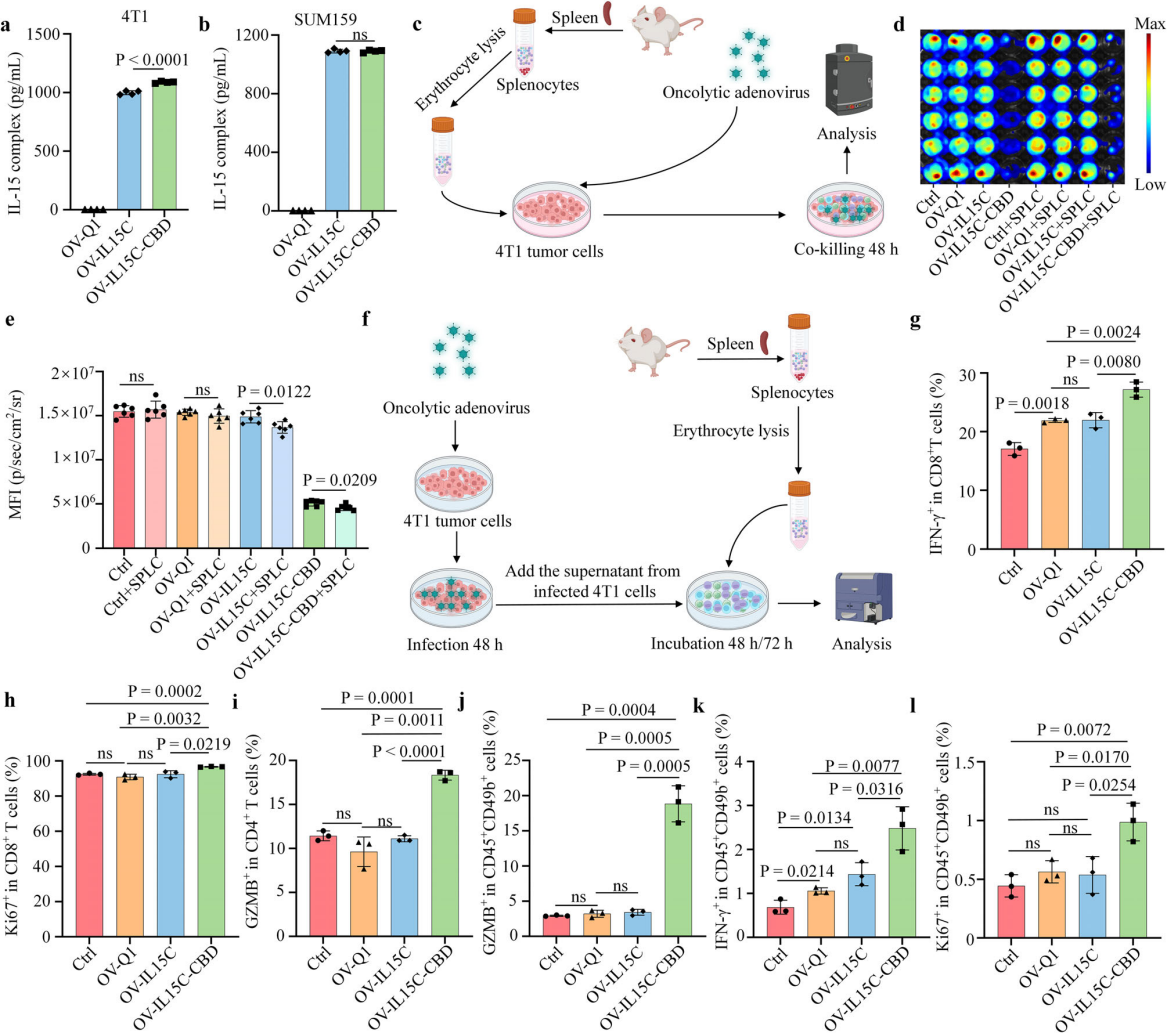
OV‐IL15C‐CBD potentiates immune activation and cytotoxicity via IL15C‐CBD immunocytokine secretion. Concentrations of IL‐15 complexes in the supernatants of 4T1 (a) and SUM159 (b) cells at 48 h post‐infection with different oncolytic adenoviruses at an MOI of 10 (n = 4/group). (c) Schematic of the oncolytic adenovirus and splenocyte co‐culture assay against 4T1 tumor cells. (d) Representative luciferase images of 4T1 tumor cells after different treatments. (e) Quantification of the average luciferase luminescence intensity in 4T1 cells across treatment groups (SPLC, splenocytes; n = 6/group). (f) Workflow for splenocyte stimulation. Supernatants from 4T1 cells treated as above (untreated, OV‐Q1, OV‐IL15C, and OV‐IL15C‐CBD) were collected at 48 h and incubated with splenocytes. Flow cytometric analysis of activated immune subsets showing CD8^+^IFN‐γ^+^ (g), CD8^+^Ki67^+^ (h), CD4^+^GZMB^+^ (i), CD45^+^CD49b^+^GZMB^+^ (j), CD45^+^CD49b^+^IFN‐γ^+^ (k), and CD45^+^CD49b^+^Ki67^+^ (l) cells (n = 3/group). The data are presented as the mean ± SD. Statistical significance was assessed using two‐way ANOVA with Tukey's multiple comparisons (e), and one‐way ANOVA with Tukey's multiple comparisons (a, b, g‐l). ns indicates no significant difference. Image created with BioRender. com, with permission (c, f).

Having identified IL15C‐CBD as a structurally and functionally optimized immunocytokine, we next assessed its capacity for virus‐mediated expression and immune activation in tumor cells. We infected 4T1 and SUM159 cells with OV‐Q1, OV‐IL15C, or OV‐IL15C‐CBD at an MOI of 10. OV‐IL15C and OV‐IL15C‐CBD exhibited comparably robust levels of IL15C secretion. In 4T1 cells, the IL15C concentration reached 999 ± 17.12 pg/mL for OV‐IL15C and 1088 ± 7.59 pg/mL for OV‐IL15C‐CBD, respectively (Figure 3a). Similarly, in SUM159 cells, the IL15C secretion levels were 1091 ± 12.64 pg/mL and 1090 ± 8.14 pg/mL, respectively (Figure 3b). These findings indicate that engineering the adenovirus to express IL15C‐CBD does not impair its capacity for IL15C secretion, and that IL15C‐CBD secretion levels are comparable to those of OV‐IL15C in both murine and human tumor cell lines.

We next assessed the biological activity of the secreted cytokine using an immune cell‐mediated tumor‐killing assay. Accordingly, we established a co‐culture system in which 4T1‐luciferase tumor cells were incubated with murine splenocytes and infected with OV‐Q1, OV‐IL15C, or OV‐IL15C‐CBD at an MOI of 2 (Figure 3c). The experimental groups consisted of splenocytes alone, oncolytic adenovirus alone, and oncolytic adenovirus combined with splenocytes. After 48 h, the reduction in tumor cell viability was quantified by measuring the mean luciferase luminescence intensity. Neither splenocytes alone nor the combination of splenocytes with OV‐Q1 further reduced cell viability, as reflected by unchanged luciferase intensity relative to the control group, indicating that nonactivated splenocytes exerted limited cytotoxicity in this system. In contrast, compared with the respective oncolytic adenoviruses alone, both OV‐IL15C and OV‐IL15C‐CBD significantly reduced luciferase luminescence intensity (by 8% and 10%, respectively), demonstrating effective IL15C‐mediated tumor cell killing induced by IL15C secretion from both vectors. Notably, OV‐IL15C‐CBD and OV‐IL15C showed comparable efficacy in reducing tumor cell viability via immune‐mediated cytotoxicity (Figure 3d,e), suggesting that the engineering of OV‐IL15C‐CBD did not compromise either its secretion or its immunostimulatory function.

Given that OV‐induced tumor cell death can itself shape the immune microenvironment, we next measured the release of high mobility group box 1 (HMGB1) as an indicator of immunogenic cell death in OV‐infected 4T1 cells. At 48 h post‐infection at an MOI of 2, supernatant HMGB1 levels in OV‐IL15C‐CBD‐treated cultures were approximately 2.4‐fold higher than those in OV‐Q1‐treated cells, while OV‐IL15C‐treated cultures showed a more modest 1.3‐fold elevation (Figure S22), suggesting that CBD fusion augments the immunogenic cell death response.

We further characterized the immunostimulatory capacity of OV‐IL15C‐CBD by analyzing conditioned media from infected tumor cells (Figure 3f). Supernatants from OV‐IL15C‐infected cultures exhibited immune activation generally comparable to that observed with OV‐Q1‐treated cultures (Figure 3g–l). In contrast, conditioned media from OV‐IL15C‐CBD‐infected cultures, which contain both secreted IL15C‐CBD immunocytokine and elevated HMGB1, induced markedly enhanced immune activation compared with those from OV‐IL15C cultures. Quantitative analysis revealed that CD8^+^ T cells exposed to these conditioned media exhibited a 1.2‐fold IFN‐γ production (Figure 3g, Figure S23a,b), accompanied by increased proliferation rates (Figure 3h, Figure S24a,b). CD4^+^ T cells also exhibited a 1.7‐fold increase in GZMB secretion (Figure 3i, Figure S25a,b). NK cells exhibited an even more pronounced response to the OV‐IL15C‐CBD‐conditioned media compared with that from OV‐IL15C controls, with a 5.5‐fold increase in GZMB production (Figure 3j, Figure S26a, b) and a 1.7‐fold greater increase in IFN‐γ secretion (Figure 3k, Figure S27a,b). This robust activation was further evidenced by a 1.8‐fold increase in NK cell proliferation (Figure 3l, Figure S28a,b). Notably, co‐incubation of OV‐IL15C‐CBD‐infected supernatants with type I collagen, type III collagen, or both in combination did not significantly reduce, and in some conditions marginally enhanced, NK cell GZMB secretion (Figure S29a–d), indicating that collagen engagement preserves the immunostimulatory activity of secreted IL15C‐CBD within a collagen‐rich microenvironment. The superior immune activation elicited by OV‐IL15C‐CBD‐conditioned media thus reflects a convergence of direct IL15C‐mediated cytokine signaling and HMGB1‐driven danger signaling, collectively establishing a proinflammatory milieu that amplifies effector responses in both CD8^+^ T cells and NK cells and underscores the multifaceted immunostimulatory mechanism of OV‐IL15C‐CBD.

### 2.4 OV‐IL15C‐CBD Remodels the Immunosuppressive TME and Drives Potent Antitumor Immunity In Vivo

**FIGURE 4:**
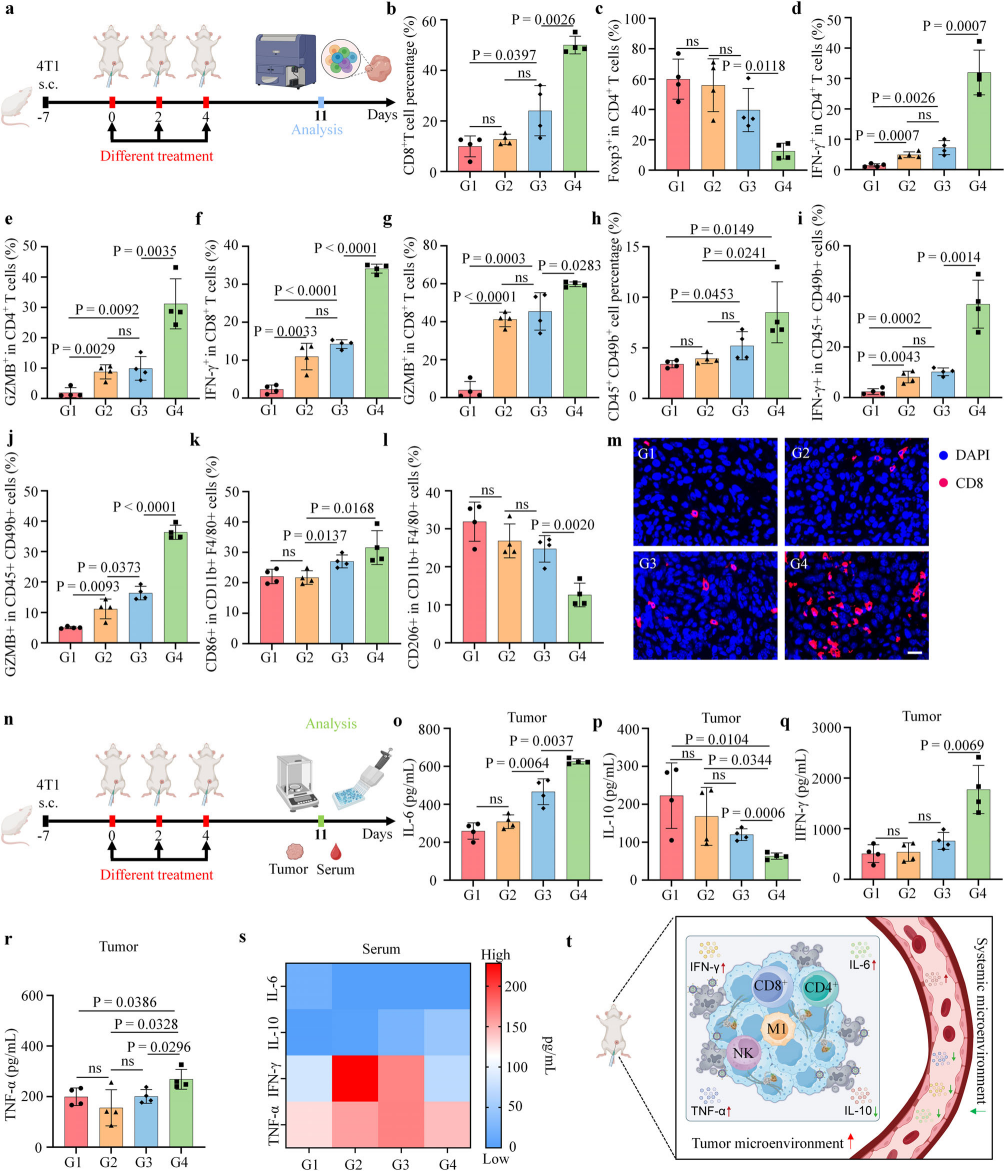
OV‐IL15C‐CBD reprograms the tumor immune microenvironment in 4T1 TNBC syngeneic models. (a) Schematic of the treatment groups and immune profiling workflow. Flow cytometric analysis of the intratumoral immune cell populations, including CD8^+^ T cells (b), CD4^+^Foxp3^+^ T cells (c), CD4^+^IFN‐γ^+^ T cells (d), CD4^+^GZMB^+^ T cells (e), CD8^+^IFN‐γ^+^ T cells (f), CD8^+^GZMB^+^ T cells (g), CD45^+^CD49b^+^ cells (h), CD45^+^CD49b^+^IFN‐γ^+^ cells (i), CD45^+^CD49b^+^GZMB^+^ cells (j), CD11b^+^F4/80^+^CD86^+^ TAMs (k), and CD11b^+^F4/80^+^CD206^+^ TAMs (l) (n = 4 mice/group). (m) Representative immunofluorescence staining of CD8^+^ T cells in tumor tissues from different treatment groups (n = 3 mice/group). Scale bar, 20 µm. (n) Experimental workflow for cytokine quantification in tumor tissues and serum via ELISA across treatment groups in the 4T1 breast cancer model. Intratumoral concentrations of IL‐6 (o), IL‐10 (p), IFN‐γ (q), and TNF‐α (r) (n = 4 mice/group). (s) Heatmap illustrating serum levels of IL‐6, IL‐10, IFN‐γ, and TNF‐α across treatment groups (n = 4 mice/group). (t) Schematic summary of cytokine profiles within the TME and systemic circulation following intratumoral OV‐IL15C‐CBD administration in 4T1 tumor‐bearing mice. G1, PBS; G2, OV‐Q1; G3, OV‐IL15C; G4, OV‐IL15C‐CBD. The data are presented as the mean ± SD. Statistical significance was assessed by one‐way ANOVA with Tukey's multiple comparisons test (b–l, o–r). ns indicates no significant difference. Image created with BioRender. com, with permission (t).

Building upon its potent immunostimulatory effects observed in vitro, we next evaluated the therapeutic efficacy of OV‐IL15C‐CBD in vivo. The 4T1 syngeneic breast tumor model was selected as the main model because it recapitulates several clinically relevant features of human TNBC, including aggressive tumor growth, poor immunogenicity, immunosuppressive immune infiltration, and prominent stromal collagen deposition. Moreover, its establishment in immunocompetent BALB/c mice enables evaluation of both oncolytic virus‐mediated tumor killing and therapy‐induced immune remodeling. Given the collagen‐dependent design of IL15C‐CBD, this collagen‐rich model provides a relevant platform for assessing tumor‐matrix‐anchored cytokine delivery. Tumors were established with 4T1 breast cancer cells. When the tumors reached 100 mm^3^, the animals were randomly assigned to receive intratumoral injections of PBS (control), OV‐Q1, OV‐IL15C, or OV‐IL15C‐CBD (Figure 4a). Comprehensive immune profiling conducted 7 days post‐treatment revealed a dramatic increase in tumor‐infiltrating CD8^+^ T cells following OV‐IL15C‐CBD administration (50.02 ± 3.48%), which significantly surpassed the levels observed in the OV‐IL15C (24.04 ± 9.93%), OV‐Q1 (12.72 ± 2.08%), and PBS (9.96 ± 4.13%) groups (Figure 4b, Figure S30a,b). Immunofluorescence staining of tumor sections further corroborated these findings, demonstrating markedly enhanced CD8^+^ T cell infiltration following OV‐IL15C‐CBD treatment (Figure 4m). Whole‐section immunofluorescence scanning further revealed that, whereas CD8^+^ T cells in the OV‐IL15C group remained largely confined to peritumoral regions, OV‐IL15C‐CBD treatment drove their pronounced redistribution into the stromal compartment, with CD8^+^ T cells penetrating deeply throughout the tumor parenchyma (Figure S31). This suggests that collagen‐anchored retention of IL15C‐CBD facilitates CD8^+^ T cell access to otherwise immune‐excluded stromal niches. Together, these data indicate that OV‐IL15C‐CBD effectively reshapes the local immune landscape by promoting the accumulation of cytotoxic T lymphocytes within the tumor.

Given that durable antitumor immunity requires not only effector T cell activation but also the alleviation of immunosuppressive constraints, we next investigated the impact of OV‐IL15C‐CBD on Tregs, whose intratumoral accumulation represents a hallmark of immune evasion in TNBC. Flow cytometric analysis revealed that compared with the control treatment, OV‐IL15C‐CBD treatment reduced intratumoral Treg infiltration to 12.58 ± 5.20%, representing a 4.8‐fold decrease relative to the PBS control (59.99 ± 13.22%) and a significant reduction compared with both the OV‐Q1 (55.99 ± 17.40%) and OV‐IL15C (39.61 ± 14.23%) treatments (Figure 4c, Figure S30c). Concomitant with Treg depletion, OV‐IL15C‐CBD also significantly increased effector T cell functionality. The proportions of IFN‐γ^+^CD4^+^ T cells and GZMB^+^CD4^+^ T cells were 4.4‐fold and 3.1‐fold greater, respectively, than those in the OV‐IL15C treatment group (Figure 4d,e; Figure S30d,e), while the numbers of IFN‐γ^+^CD8^+^ T cells and GZMB^+^CD8^+^ T cells were 2.4‐fold and 1.3‐fold greater, respectively (Figure 4f,g; Figure S30f,g). These results highlight the unique capacity of IL15C‐CBD to reprogram the TNBC immune landscape toward effective antitumor immunity.

The immunomodulatory effects extended to the innate immune population, and a marked increase in NK (CD45^+^CD49b^+^) cell infiltration was observed following OV‐IL15C‐CBD treatment (8.52 ± 3.01% versus 5.21 ± 1.39% in OV‐IL15C controls, Figure 4h; Figure S32a,b). NK cell cytotoxicity was also markedly enhanced following OV‐IL15C‐CBD treatment, as evidenced by 3.6‐fold and 2.2‐fold increases in IFN‐γ^+^ and GZMB^+^ NK cells, respectively (Figure 4i,j; Figure S32c,d). Concurrently, OV‐IL15C‐CBD induced a fundamental repolarization of TAMs, increasing proinflammatory M1‐like (CD11b^+^F4/80^+^CD86^+^) populations while decreasing immunosuppressive M2‐like (CD11b^+^F4/80^+^CD206^+^) subsets (Figure 4k,l; Figure S33a–c). OV‐IL15C‐CBD establishes a self‐reinforcing immunostimulatory milieu within the TME, thereby coordinately remodeling adaptive and innate immunity to drive synergistic proinflammatory responses.

Cytokine profiling of tumor lysates revealed this localized immune activation, with significant increases in IL‐6 (1.3‐fold), IFN‐γ (2.3‐fold), and TNF‐α (1.3‐fold) levels, coupled with a 1.9‐fold reduction in immunosuppressive IL‐10 levels compared with those in OV‐IL15C controls (Figure 4n–r). In contrast, serum cytokine measurements showed that OV‐IL15C‐CBD did not induce an obvious systemic proinflammatory cytokine elevation. IL‐6 levels remained very low or undetectable across groups, while circulating IFN‐γ and TNF‐α levels remained comparable between the PBS and OV‐IL15C‐CBD groups. Notably, serum levels of the anti‐inflammatory cytokine IL‐10 were moderately elevated in the OV‐IL15C‐CBD group, reflecting a compensatory immunoregulatory response that maintains systemic immune homeostasis and precludes a proinflammatory cytokine surge (Figure 4s). These results indicate that tumor‐localized IL15C‐CBD delivery effectively ameliorates tumor‐associated systemic immune dysregulation (Figure 4t). Together, these findings demonstrate that OV‐IL15C‐CBD orchestrates a profound remodeling of the tumor immune microenvironment, establishing a self‐reinforcing immunostimulatory milieu that promotes effector T and NK cell responses, repolarizes TAMs, and sustains a robust local proinflammatory network while mitigating the risk of systemic inflammation and immune‐related toxicity.

### 2.5 OV‐IL15C‐CBD Treatment Induces Robust Immune Activation Gene Signatures in the TME

**FIGURE 5:**
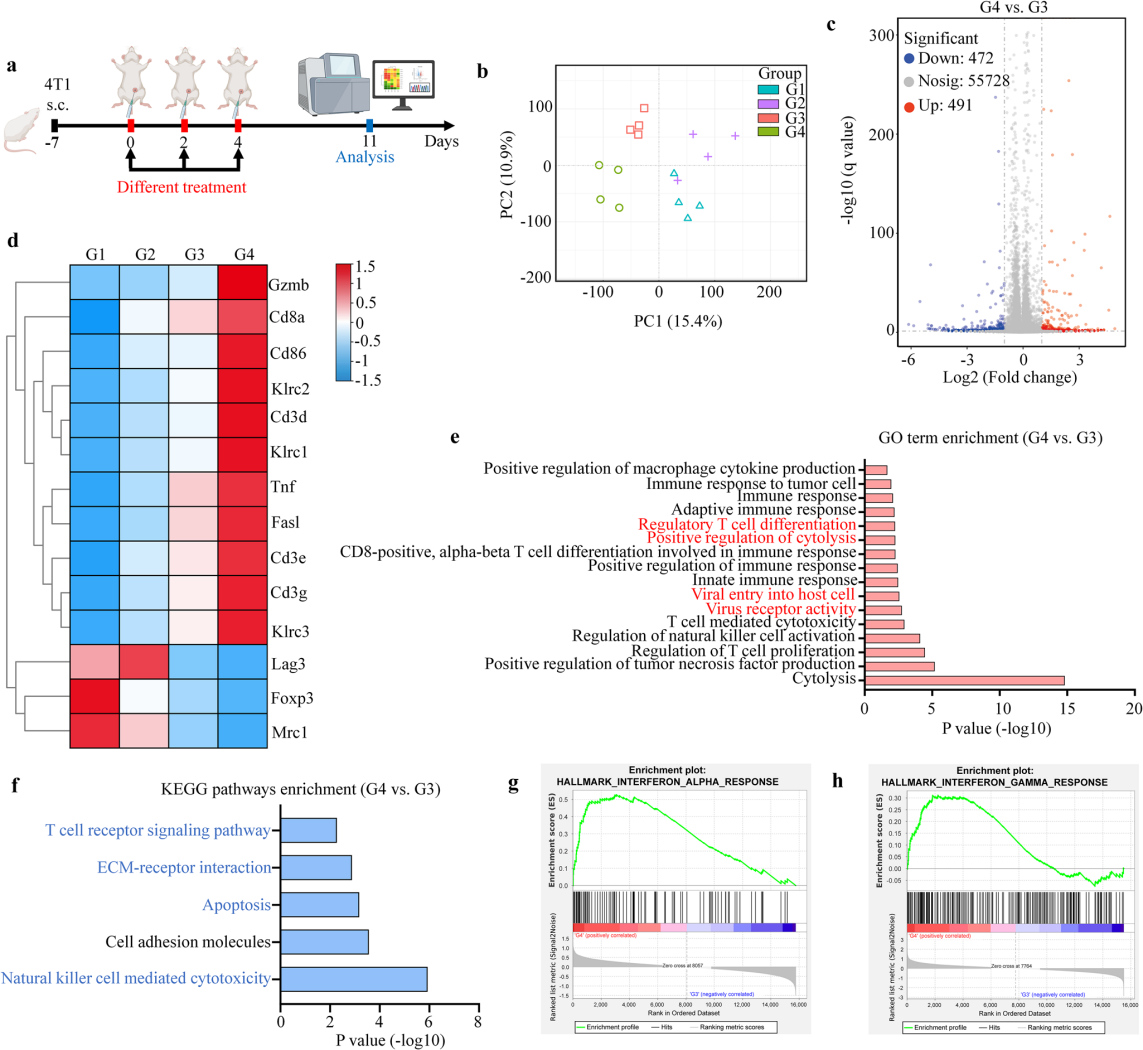
Transcriptomic profiling reveals OV‐IL15C‐CBD‐mediated immune reprogramming in 4T1 tumors. (a) Experimental workflow for RNA sequencing analysis of 4T1 tumors in mice following treatment with PBS (G1), OV‐Q1 (G2), OV‐IL15C (G3), or OV‐IL15C‐CBD (G4). (b) PCA demonstrating distinct clustering of treatment groups. (c) Volcano plot of differentially expressed genes between OV‐IL15C‐CBD and OV‐IL15C treatments. (d) Heatmap of immune‐related gene expression profiles across treatment groups. Functional enrichment analysis of differentially expressed genes between OV‐IL15C‐CBD and OV‐IL15C, showing enrichment pathways identified by GO (e), KEGG (f), and GSEA (g, h). n = 4 mice/group. Image created with BioRender. com, with permission (a).

We performed transcriptomic profiling of 4T1 tumors following OV‐IL15C‐CBD treatment to elucidate the molecular mechanisms underlying the observed immune activation (Figure 5a). Principal component analysis (PCA) revealed distinct clustering of the treatment groups, demonstrating strategy‐specific transcriptional responses (Figure 5b). Comparative analysis indicated that PBS‐treated and OV‐Q1‐treated tumors maintained an immunosuppressive signature characterized by elevated expression of immune checkpoint molecules (Lag3), regulatory T cell markers (Foxp3), and M2 macrophage‐associated genes (Mrc1), coupled with suppressed expression of cytotoxic effector genes (Cd8a, Cd86, and the NK cell receptor Klrc1) and cytolytic mediators (Tnf and Gzmb) (Figure 5d). In contrast, OV‐IL15C‐CBD therapy fundamentally reprogrammed the tumor transcriptome to establish a self‐reinforcing immunostimulatory milieu. This persistent immune activation was evidenced by the sustained upregulation of IL‐15 signaling cascades that drive the proliferation and activation of cytotoxic T and NK cells, thereby amplifying local antitumor immunity.

Differential gene expression analysis identified 491 genes upregulated in OV‐IL15C‐CBD‐treated tumors compared with OV‐IL15C control tumors (Figure 5c). Gene Ontology (GO) analysis revealed that these genes were predominantly enriched in immune effector processes, including regulatory T cell differentiation, positive regulation of cytolysis, viral entry into host cell, and virus receptor activity (Figure 5e). This indicates that OV‐IL15C‐CBD elicits a broad transcriptional response encompassing both immune activation and virus‐host interaction pathways within the TME.

Kyoto Encyclopedia of Genes and Genomes (KEGG) analysis further demonstrated broad enrichment in pathways critical for antitumor immunity, including the T cell receptor signaling pathway, apoptosis, NK cell‐mediated cytotoxicity, and ECM‐receptor interaction (Figure 5f), highlighting the breadth of immune remodeling mediated by OV‐IL15C‐CBD. Gene set enrichment analysis (GSEA) revealed significant activation of the IFN‐α and IFN‐γ signaling pathways in OV‐IL15C‐CBD‐treated tumors compared with OV‐IL15C controls (Figure 5g,h). This robust activation of the IFN pathway not only drives direct apoptosis of tumor cells but also potentiates effector immune cell responses, thereby establishing a proinflammatory milieu that sustains antitumor immunity. These findings demonstrate that OV‐IL15C‐CBD orchestrates a multilayered immunogenic shift in the TME, overcoming immunosuppressive barriers and activating both innate and adaptive antitumor immune responses through transcriptional reprogramming.

### 2.6 Increased Antitumor Efficacy of OV‐IL15C‐CBD via Improved Tumor Retention

**FIGURE 6:**
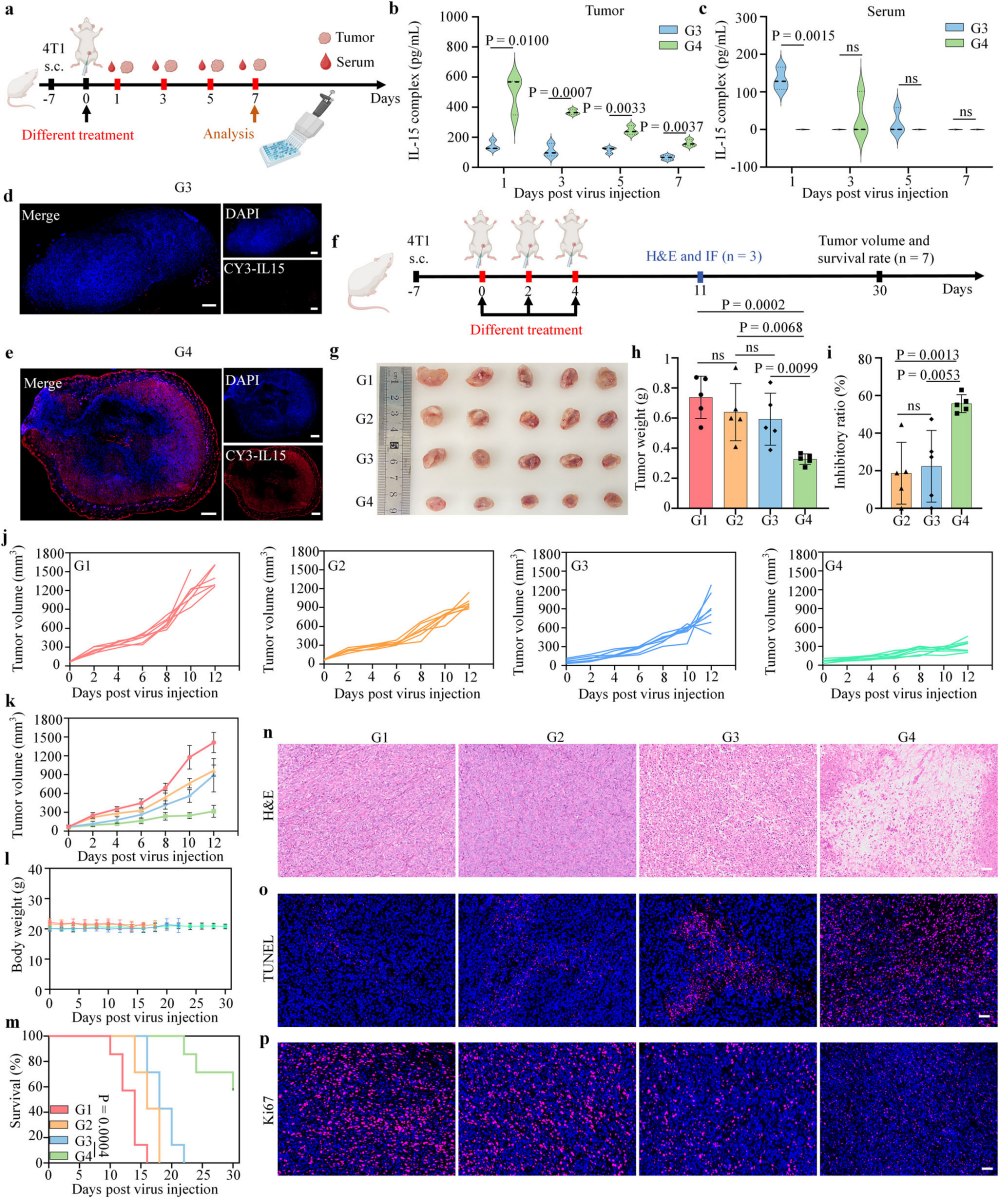
Therapeutic efficacy of OV‐IL15C‐CBD in 4T1 tumor‐bearing mice. (a) Experimental timeline for the analysis of the distribution of IL15C in tumor tissues and serum following a single dose of OV‐IL15C or OV‐IL15C‐CBD in the 4T1 breast cancer model. ELISA quantification of IL15C in tumor tissues (b) and serum (c) at the indicated time points (n = 3 mice/group). Representative immunofluorescence imaging of IL‐15 localization in tumor tissues 24 h after treatment with OV‐IL15C (d) or OV‐IL15C‐CBD (e) (n = 3 mice/group). Scale bar, 500 µm. (f) Experimental design for evaluating in vivo therapeutic efficacy. (g) Images of tumors excised on day 7 after the final treatment. (h) Changes in tumor weight in response to different treatments (n = 5 mice/group). (i) Tumor inhibition rates for each treatment group (n = 5 mice/group). Individual (j) and mean tumor growth curves (k) for each treatment group (n = 7 mice/group). (l) Body weight monitoring during treatment (n = 7 mice/group). (m) Kaplan‐Meier survival curves of 4T1 tumor‐bearing mice subjected to different treatments (n = 7 mice/group). (n) Representative H&E staining of tumor tissues from different treatment groups (n = 3 mice/group). Scale bar, 50 µm. (o) Representative TUNEL staining of tumor tissues from different treatment groups (n = 3 mice/group). (p) Representative Ki67 staining of tumor tissues from different treatment groups (n = 3 mice/group). Treatment groups included G1 (PBS), G2 (OV‐Q1), G3 (OV‐IL15C), and G4 (OV‐IL15C‐CBD). The data are presented as the mean ± SD. Statistical significance was assessed using two‐way ANOVA with Tukey's multiple comparisons (b, c), one‐way ANOVA with Tukey's multiple comparisons (h, i), and the log‐rank (Mantel‐Cox) test (m). ns indicates no significant difference. Image created with BioRender. com, with permission (a, f).

Building on the transcriptomic findings, we next investigated whether the CBD modification enhanced the therapeutic potential of the OVs by improving intratumoral retention of the secreted immunocytokine. First, in the 4T1 breast cancer model, mice were administered a single intratumoral dose of either OV‐IL15C or OV‐IL15C‐CBD at 1 × 10^7^ PFU. Tumor and serum samples were collected at 1, 3, 5, and 7 days post‐treatment for quantitative analysis of IL15C distribution (Figure 6a) by ELISA, which revealed that OV‐IL15C‐CBD maintained significantly higher IL15C concentrations in tumor tissues at all time points. At day 7 post‐treatment, the intratumoral IL15C levels in the OV‐IL15C‐CBD group remained nearly 2.5‐fold higher than those in the OV‐IL15C group (Figure 6b), indicating sustained local retention. Notably, serum analysis revealed comparable systemic IL15C levels at most time points, except on day 1, when OV‐IL15C‐CBD‐treated tumors exhibited significantly lower circulating IL15C levels than did OV‐IL15C‐treated tumors (Figure 6c). These findings suggest that CBD‐mediated tethering to the tumor ECM reduces early systemic spillover, potentially mitigating off‐target toxicity. Immunofluorescence analysis further confirmed the broader distribution and significantly increased IL‐15 signal intensity in the OV‐IL15C‐CBD‐treated tumors (Figure 6d,e), supporting improved intratumoral accumulation. Multiplex immunofluorescence staining of tumor cryosections at 48 h post‐treatment demonstrated effective virus retention in both groups, while IL‐15 immunoreactivity in OV‐IL15C‐CBD‐treated tumors was more broadly distributed and showed extensive colocalization with type I and type III collagen throughout the stromal compartment (Figure S34a,b). This spatial enrichment of IL15C may help overcome heterogeneity and establish a self‐reinforcing immunostimulatory milieu within the TME, thereby laying the foundation for persistent and robust local immune activation.

The antitumor activity of OV‐IL15C‐CBD was subsequently evaluated in 4T1 tumor‐bearing mice to determine whether the enhanced intratumoral IL15C retention translated to improved therapeutic outcomes. The animals were randomly divided into four treatment groups: PBS (G1, control), OV‐Q1 (G2, backbone virus), OV‐IL15C (G3), and OV‐IL15C‐CBD (G4) (Figure 6f). The OV‐IL15C‐CBD treatment significantly reduced tumor growth. At 12 days after treatment, the mean tumor volumes in the PBS, OV‐Q1, OV‐IL15C, and OV‐IL15C‐CBD groups were 1411.9, 965.4, 890.0, and 316.1 mm^3^, respectively (Figure 6j,k). Consistent with these findings, the weights of the tumors excised 7 days after the final treatment were approximately 56% lower in the OV‐IL15C‐CBD group than in the PBS group (Figure 6g,h), with inhibition rates 3‐fold and 2.5‐fold greater than those achieved by OV‐Q1 and OV‐IL15C, respectively (Figure 6i). Although no complete tumor regression was observed by day 30 in any group, OV‐IL15C‐CBD treatment meaningfully extended the median survival to more than 30 days, compared with 18 days for OV‐IL15C, 16 days for OV‐Q1, and 14 days for PBS (Figure 6m). These data establish a direct correlation between the pronounced survival benefit of OV‐IL15C‐CBD and its effective local cytokine enrichment, highlighting the critical contribution of the CBD domain.

We further examined the generalizability of this therapeutic advantage in the EMT6 tumor model, where immunofluorescence staining confirmed abundant stromal deposition of type I and type III collagen (Figure S35a,b), providing a mechanistically relevant context for evaluating anchoring mediated by the CBD. Consistent with the 4T1 results, OV‐IL15C‐CBD conferred markedly superior efficacy. At day 7 after the final treatment, tumor weights were reduced by approximately 80%, 69%, and 62% relative to the PBS, OV‐Q1, and OV‐IL15C controls, respectively (Figure S35c,d), yielding inhibition rates approximately 2.2‐fold and 1.7‐fold greater than those achieved by OV‐Q1 and OV‐IL15C (Figure S35e). This advantage persisted throughout the observation period, as reflected by tumor growth kinetics, with mean tumor volumes reaching 1575.0, 1123.5, 955.4, and 161.3 mm^3^ in the PBS, OV‐Q1, OV‐IL15C, and OV‐IL15C‐CBD groups, respectively, at day 14 after the final treatment (Figure S35f,g). Furthermore, survival analysis revealed a median survival exceeding 30 days in the OV‐IL15C‐CBD group, compared with 20 days for OV‐IL15C, 18 days for OV‐Q1, and 14 days for PBS (Figure S36). Moreover, body weights remained stable across all treatment groups in the EMT6 tumor model (Figure S37), indicating favorable systemic tolerability. The reproducible efficacy gains across these two distinct collagen‐rich models indicate that stromal anchoring mediated by the CBD is central to the therapeutic enhancement, rather than being specific to a single model.

Histopathological evaluation confirmed both the therapeutic efficacy and biosafety profile of OV‐IL15C‐CBD. H&E staining revealed extensive tumor necrosis and marked disruption of tumor architecture in OV‐IL15C‐CBD‐treated specimens (Figure 6n). TUNEL immunofluorescence analysis revealed a marked increase in the number of apoptotic tumor cells (Figure 6o), and Ki67 immunofluorescence analysis indicated significant inhibition of tumor cell proliferation in the OV‐IL15C‐CBD group (Figure 6p). Safety assessments revealed no treatment‐related pathological alterations in major organs (heart, liver, spleen, lung, or kidney) across all groups (Figure S38). Comprehensive serum biochemistry analyses, including albumin (ALB), alkaline phosphatase (ALP), alanine aminotransferase (ALT), aspartate aminotransferase (AST), total bilirubin (TBIL), direct bilirubin (DBIL), total bile acids (TBA), uric acid (UA), serum creatinine (CREA), and γ‐glutamyl transferase (γ‐GT), revealed no evidence of organ toxicity (Figure S39a–j). The body weights of the tumor‐bearing mice remained stable throughout the treatment period (Figure 6l), further supporting the absence of systemic toxicity. From a virological perspective, absolute quantification of adenoviral E1A at 48 h after a single administration revealed robust intratumoral replication for OV‐Q1, OV‐IL15C, and OV‐IL15C‐CBD, with no significant differences observed among the three viral groups (Figure S40a). Crucially, E1A copy numbers in the liver (Figure S40b), spleen (Figure S40c), lung (Figure S40d), and kidney (Figure S40e) of mice receiving these viruses remained indistinguishable from the PBS control, verifying the strict confinement of viral amplification to the tumor compartment without aberrant systemic dissemination. These findings demonstrate that OV‐IL15C‐CBD achieves potent antitumor effects through direct oncolysis and immune activation while maintaining an excellent safety profile with no detectable off‐target toxicity, underscoring its translational potential for cancer immunotherapy.

## 3 Discussion

We engineered an oncolytic adenovirus encoding a collagen‐binding IL‐15 superagonist to overcome the physical and immunological barriers characteristic of TNBC, thereby transforming the dense, immunosuppressive tumor stroma into a self‐reinforcing, immunostimulatory milieu. TNBC lesions are characterized by densely cross‐linked collagen fibers and aligned stromal architectures that impede cytotoxic lymphocyte infiltration and engage inhibitory immune receptors, thereby reinforcing immune exclusion [22, 30]. The TME is dominated by TAMs and myeloid‐derived suppressor cells (MDSCs), with a marked deficiency of effector T and NK cells, features closely associated with resistance to immune checkpoint blockade [31]. By anchoring IL‐15 within the collagen‐rich ECM, OV‐IL15C‐CBD remodeled the TME into a localized proinflammatory niche. This facilitated the deep infiltration of CD8^+^ T cells into stromal compartments that are typically resistant to lymphocyte access and drove a phenotypic shift in myeloid cells toward an M1‐like proinflammatory state. Coordinated innate and adaptive immune activation reprogrammed immune‐cold tumors into permissive niches that support sustained antitumor immunity. These results reveal that stromal repurposing through ECM‐tethered cytokine delivery dismantles structural and immunosuppressive barriers to lymphocyte access, offering a rational strategy to overcome a central mechanism of immunotherapy resistance in TNBC and other stroma‐rich malignancies.

A defining feature of OV‐IL15C‐CBD therapy is the robust intratumoral expansion and activation of cytotoxic lymphocytes. Treated TNBC tumors became densely populated with CD8^+^ T cells and NK cells that displayed activation markers, including high levels of IFN‐γ and GZMB, indicative of potent effector function. This immune cell recruitment is particularly noteworthy given that untreated TNBCs often lack significant T cell infiltration despite their antigenicity [32, 33]. In our study, CD8^+^ T cell enrichment was accompanied by a marked increase in the CD8^+^ T cell‐to‐Treg ratio, shifting the balance toward antitumor immunity. Clinically, a high ratio of cytotoxic T cells to Tregs is associated with improved immunotherapy responses and patient outcomes [34]. Thus, by promoting sustained intratumoral delivery of IL‐15, OV‐IL15C‐CBD created a milieu permissive to tumor rejection. In addition to mobilizing lymphocytes, OV‐IL15C‐CBD therapy instigated a pronounced shift in the myeloid cell landscape toward a proinflammatory state. Treated tumors showed evidence of TAMs adopting M1‐like characteristics and a reduction in immunosuppressive myeloid populations. This reprogramming is critical because immunotherapy resistance in TNBC is strongly associated with an overabundance of M2‐polarized macrophages and immature myeloid cells that suppress T‐cell activity [35]. Although IL‐15 primarily acts on NK and T cells, the robust IFN‐γ release and inflammatory signaling by these lymphocytes can drive macrophage polarization toward a classically activated, tumoricidal phenotype [36]. Indeed, in our study, the innate and adaptive arms of the immune system appeared to reinforce each other. Activated CD8^+^ T cells and NK cells secreted cytokines that further activated macrophages, while proinflammatory macrophages secreted chemokines that attracted and stimulated more T cells, creating a positive feedback loop. Such reciprocal crosstalk maintains a self‐reinforcing immune milieu within the tumor.

The key advantage of OV‐IL15C‐CBD lies in anchoring IL‐15 within the tumor stroma, preventing its rapid diffusion into the circulation and avoiding the severe systemic toxicities associated with conventional cytokine therapy. Systemically administered cytokines such as high‐dose IL‐2 produce potent antitumor responses but are notorious for life‐threatening side effects, including capillary leak syndrome and multi‐organ damage [37, 38]. Even engineered IL‐15 superagonists such as N‐803 require systemic dosing and can provoke off‐target immune expansion [39]. In the 4T1 model, tumor progression is typically accompanied by systemic inflammation, extensive extramedullary hematopoiesis, and robust MDSC expansion, which together drive dramatic splenomegaly [40]. IL‐15 can counteract this myeloid skewing by decreasing MDSC accumulation and restoring physiological hematopoiesis [41]. Crucially, the CBD in OV‐IL15C‐CBD concentrates IL‐15 within the TME, greatly enhancing local retention and limiting peripheral cytokine dissemination. This spatial confinement blunts the systemic inflammatory cues that fuel extramedullary hematopoiesis and splenic hypertrophy while focusing immunostimulatory effects on the tumor. OV‐IL15C‐CBD thus delivers the full immunostimulatory capacity of IL‐15 locally with minimal off‐target toxicity to normal tissues.

Many solid tumors possess dense stromal structures that both hinder immune cell infiltration and restrict cytokine diffusion [7]. In such fibrotic, collagen‐rich microenvironments, effector immune cells are often trapped within stromal regions surrounding the tumor, resulting in the immune‐excluded phenotype [30]. In the 4T1 TNBC model, OV‐IL15C‐CBD profoundly enhanced the intratumoral infiltration and activation of CD8^+^ T cells, effectively remodeling the immune‐cold TME into an inflamed state. This demonstrates that ECM‐anchored cytokines can overcome stroma‐induced immune exclusion. Given the ubiquitous presence of fibrillar collagen within the stroma of diverse solid malignancies, this localized retention strategy offers broad translational potential [42]. Many solid tumors, including breast, lung, liver, and urothelial carcinomas, contain a similar collagen‐rich stroma that tends to isolate immune cells from the tumor parenchyma [43]. By stably retaining IL‐15 within the TME, this approach enhances local immune stimulation while significantly reducing systemic toxicity. The ECM‐anchored cytokine delivery strategy, implemented via an OV platform, offers a rational therapeutic avenue for tumors with stroma‐driven immunosuppression across multiple solid tumor types.

While this study offers a promising framework for localized cytokine immunotherapy, several limitations warrant consideration. Our findings are based on the 4T1 and EMT6 murine models, which exhibit aggressive growth and systemic inflammation that are not always representative of human tumors. Translational studies will be needed to validate whether IL‐15 enrichment can similarly reprogram the human TME. An additional critical consideration concerns the host antiviral immune response elicited by repeated OV administration. Although our regimen employed a relatively low intratumoral dose of 1 × 10^7^ PFU per injection, substantially lower than the 10^8–^10^9^ PFU typically required in conventional murine oncolytic adenovirus models [44, 45], repeated administrations inevitably increase viral antigen exposure. This cumulative antigen load can trigger the production of neutralizing antibodies and the activation of complement cascades, which may accelerate viral clearance upon subsequent dosing [46, 47]. Such antiviral immunity could theoretically attenuate both viral genome replication and sustained IL15C‐CBD transgene expression, thereby diminishing the therapeutic benefit of repeated treatment cycles. This concern is underscored by recent conference consensus highlighting the inherent tension between antiviral and antitumor immunity as a central challenge in oncolytic virotherapy [48]. Crucially, intratumoral administration circumvents circulating neutralizing antibodies more effectively than systemic delivery, sustaining productive viral replication within the localized tumor compartment [47, 49].

Furthermore, recent advances in OV engineering offer robust solutions to this challenge. Chimeric adenoviruses constructed by swapping hexon or fiber proteins across distinct serotypes have demonstrated robust evasion of patient‐derived neutralizing antibodies while maintaining oncolytic potency [50]. Complementary strategies, including polymer‐based nanoparticle encapsulation of viral particles and alternating use of heterologous viral backbones, may further shield OVs from immune recognition and prolong the window of productive transgene expression during repeated dosing regimens [49, 51]. Future approaches may also explore incorporating advanced shielding mechanisms to delay host antiviral immune responses further. We also did not assess long‐term outcomes such as memory T cell formation or efficacy against metastatic lesions, which are important considerations for tumors such as TNBC that frequently disseminate. While OV‐IL15C‐CBD improved survival, incomplete tumor clearance suggests residual immunosuppressive pathways, highlighting the need for rational combinations, such as immune checkpoint blockade. Ultimately, although OV‐IL15C‐CBD represents a substantial advance in tumor‐localized cytokine therapy, further investigation is required to confirm efficacy across tumor types, evaluate durability, and optimize delivery.

In summary, ECM‐anchored cytokine delivery enables localized immune reprogramming and durable immune engagement. OV‐IL15C‐CBD establishes a localized cytokine reservoir that overcomes both physical and immunological barriers to immune infiltration. By transforming immune‐cold tumors into sites of sustained immune activation, this approach redefines the therapeutic potential of cytokine‐based immunotherapy. It offers a compelling strategy for treating TNBC and other stroma‐rich and treatment‐resistant malignancies.

## 4 Experimental Section

### 4.1 Cells and Animals

All cell lines used in this study were purchased from the Chinese Academy of Sciences Cell Bank, unless otherwise specified. 4T1 (RRID: CVCL_J239), EMT6 (RRID: CVCL_1923), and SUM159 (RRID: CVCL_5423) cells were cultured in RPMI‐1640 medium (Basalmedia) with 10% fetal bovine serum (FBS; NEWZERUM). HeLa (RRID: CVCL_0030), LLC (RRID: CVCL_4358), A549 (RRID: CVCL_0023), and HEK293A (RRID: CVCL_6910) cells were maintained in DMEM (Basalmedia) containing 10% FBS. Suspension‐adapted HEK293F (RRID: CVCL_6642) cells were propagated in serum‐free FreeStyle 293 medium (Union). All adherent cell lines were cultured at 37°C in a humidified 5% CO2 atmosphere. HEK293F suspension cultures were maintained in a shaker incubator at 37°C, 8% CO_2_, and 140 rpm. For authentication, all obtained cell lines were acquired from authenticated repositories and handled according to the suppliers’ recommendations. Cells were used within 3–10 passages after thawing to minimize genetic drift. All cell lines were routinely tested and confirmed to be free of mycoplasma contamination using a commercial kit (Transgen). Female BALB/c mice (6‐8 weeks old, 18–20 g) were purchased from Lingchang Biotechnology (Shanghai, China). All animal experiments were performed in compliance with the Guide for the Care and Use of Laboratory Animals and approved by the Animal Ethics Committee of Fudan University (approval No. 20250227‐179).

### 4.2 Bioinformatics Analysis

RNA sequencing data for BRCA and matched normal tissues were obtained from TCGA and GTEx projects via the UCSC Xena browser (https://xenabrowser.net/datapages/). Expression data were normalized using the Toil pipeline and transformed to log_2_ (TPM + 1) for downstream analyses. Differential expression of IL‐15 and IL‐15Rα between tumor and normal tissues was assessed accordingly. Single‐sample GSEA was performed using the GSVA R package (v1.46.0) to investigate immune‐related signatures and evaluate associations with inflammatory pathways. Immune cell infiltration scores for 24 cell types were inferred using the method of Bindea et al. [52] and correlated with IL15 and IL15RA expression across cancer types. Survival analyses, including OS and DFS, were conducted via GEPIA2 (http://gepia2.cancer‐pku.cn/#index) to evaluate the prognostic significance of IL‐15 and IL‐15Rα expression in BRCA.

### 4.3 Homology Modeling and Molecular Docking

Three‐dimensional structures of IL15C, CBD‐IL15C, and IL15C‐CBD were predicted using AlphaFold2 (v2.3.0), and only models with a mean pLDDT score above 90 were retained for further analysis. Homology models of murine IL‐15Rβ and IL‐15Rγc were generated using the SWISS‐MODEL platform (https://swissmodel.expasy.org/), based on the structural templates PDB:6DG5 and UniProt: P34902, respectively. Protein‐protein docking was conducted using the HawkDock server (http://cadd.zju.edu.cn/hawkdock/) to evaluate interactions between IL15C variants and either IL‐15Rβ or IL‐15Rγc. For each complex, binding free energies were estimated in triplicate using the molecular mechanics/generalized Born surface area (MM/GBSA) approach [53].

### 4.4 Plasmid Construction and Recombinant Protein Expression

The IL15C construct was engineered by fusing murine IL‐15 to the sushi domain of IL‐15Rα via a flexible (GGGS)_3_ linker. The codon‐optimized sequence was synthesized by GENEWIZ (Suzhou, China) and subcloned into the gWIZ eukaryotic expression vector, which includes an N‐terminal signal peptide and a C‐terminal 6 × His tag. To generate IL15C‐CBD, a sequence encoding the CBD protein (A3 domain of von Willebrand factor [54]) was fused to the C‐terminus of IL15C via a (GGGS)_2_ linker. A standalone gWIZ‐CBD expression plasmid was additionally constructed by subcloning the CBD coding sequence into the gWIZ vector with a C‐terminal 6 × His tag, enabling independent production of the CBD protein for binding validation experiments. Codon‐optimized sequences encoding the extracellular domains of murine IL‐15Rβ and IL‐15Rγc were similarly synthesized and inserted into separate gWIZ vectors, each containing an N‐terminal secretion signal and a C‐terminal Fc fusion. All recombinant proteins were expressed in HEK293F cells adapted to suspension culture in serum‐free FreeStyle 293 medium. Cells were seeded at a density of 1 × 10^6^ cells/mL and transfected with the respective plasmids using 40 kDa linear polyethyleneimine (PEI). After 7 days of incubation, culture supernatants were harvested, and proteins were purified by affinity chromatography following established protocols [55, 56]. Protein purity and molecular weight were assessed by SDS‐PAGE, and concentrations were measured using a BCA protein assay. The specificity was confirmed by immunoblotting with an anti‐IL‐15 primary antibody (Abcam) and an HRP‐conjugated secondary antibody (Servicebio), following standard Western blot procedures.

### 4.5 Physicochemical Characterization

IL15C and IL15C‐CBD proteins were diluted in phosphate‐buffered saline (PBS, pH 7.4) to a final concentration of 2 µg/mL and then passed through 0.22 µm sterile filters. DLS and electrophoretic light scattering were performed at 25°C using a Zetasizer Nano ZS instrument (Malvern Instruments, UK) to determine particle size distribution and zeta potential, respectively. All measurements were conducted in triplicate, and data were processed using Zetasizer software (Malvern).

### 4.6 ELISA‐Based Binding Assays

High‐binding 96‐well plates (Corning) were coated overnight at 4°C with IL‐15Rβ‐Fc or IL‐15Rγc‐Fc (5 µg/mL each), type I collagen (10 µg/mL; EMD Millipore), or type III collagen (20 µg/mL; EMD Millipore). After washing with PBST (PBS supplemented with 0.05% Tween‐20), wells were incubated with IL15C or IL15C‐CBD at graded concentrations (2, 10, or 50 µg/mL) for 1 h at 37°C. In parallel, purified CBD protein was independently evaluated at the same concentration range (2, 10, and 50 µg/mL) against plates coated with type I collagen (10 µg/mL), IL‐15Rβ‐Fc (5 µg/mL), or IL‐15Rγc‐Fc (5 µg/mL) to confirm the binding specificity of the CBD moiety. Following additional washes, plates were incubated for 1 h at 37°C with horseradish peroxidase (HRP)‐conjugated mouse anti‐His tag antibody (1:3000; Absin). After a final wash, TMB substrate solution (Beyotime) was added and allowed to react in the dark for 30 min at room temperature. The reaction was stopped with 2 M H_2_SO_4_, and absorbance was recorded at 450 nm using a microplate reader (Bio‐Rad).

### 4.7 Tissue Collagen Characterization and Ex Vivo Binding Assays

Stromal collagen architecture across distinct tissue types was characterized by subjecting formalin‐fixed paraffin‐embedded sections of 4T1 tumors, EMT6 tumors, mouse tail, and mouse skin to immunofluorescence staining for type I and type III collagen with DAPI counterstaining. The spatial anchoring of the engineered immunocytokine within the TME was evaluated by applying purified IL15C and IL15C‐CBD proteins (50 µg/mL each) to cryosections of 4T1 tumor tissues, followed by overnight incubation at 4°C. Sections were subsequently co‐stained with antibodies against type I collagen, type III collagen, and the His tag to visualize protein localization relative to stromal collagen fibers. Slide scanning and analysis were performed using the Olympus SLIDEVIEW VS200 research slide scanner.

### 4.8 Packaging and Purification of Engineered Oncolytic Adenoviruses

Gene sequences encoding IL15C or IL15C‐CBD, each containing an N‐terminal signal peptide, were PCR‐amplified and subcloned downstream of the mCMV promoter in the pENTR‐EGFP shuttle vector, which harbors the hTERT‐E1A‐IRES‐E1B‐mCMV‐IRES‐EGFP cassette. The resulting shuttle constructs were recombined with the pAd adenoviral backbone via LR Clonase‐mediated recombination, as previously described [57], generating pAd‐EGFP, pAd‐IL15C‐EGFP, and pAd‐IL15C‐CBD‐EGFP. Recombinant adenoviral plasmids were transfected into HEK293A cells at 30% confluence using 40 kDa linear PEI in serum‐free DMEM. After 6–8 h, the medium was replaced with DMEM supplemented with 1% FBS, and cells were cultured at 37°C in 5% CO_2_ until cytopathic effects were observed. Cells and supernatants were harvested, and viral particles were released through three cycles of freeze‐thaw lysis. Primary viral stocks (designated OV‐Q1, OV‐IL15C, and OV‐IL15C‐CBD) were subsequently amplified in suspension‐adapted HEK293F cells. Purification of recombinant adenoviruses was carried out by cesium chloride gradient ultracentrifugation (Beckman, 35 000 rpm, 4°C, 2 h). Purified virions were resuspended in PBS. Viral titers were quantified by TCID_50_ assays in HeLa cells, using EGFP fluorescence as a reporter of infection efficiency.

### 4.9 Assessment of the Infectivity of Engineered Oncolytic Adenoviruses

To evaluate infectivity, 4T1, EMT6, and SUM159 cells were seeded in 96‐well plates at a density of 8 × 10^3^ cells/well and allowed to adhere overnight. Cells were then infected with OV‐Q1, OV‐IL15C, or OV‐IL15C‐CBD at an MOI of 1. After 2 h of incubation, the medium was replaced with fresh culture medium containing 1% FBS, and cells were cultured for an additional 48 h. EGFP fluorescence was visualized using a BZ‐X series fluorescence microscope (Keyence). For quantitative analysis, tumor cells were seeded in 6‐well plates at a density of 1 × 10^6^ cells/well and infected under identical conditions. After 48 h, cells were harvested, washed twice with PBS, and analyzed by flow cytometry (BD Biosciences) to quantify EGFP‐positive populations. Data were processed using FlowJo software (v10.9.0).

### 4.10 Cell Viability Assay

4T1, EMT6, SUM159, LLC, and A549 cells were seeded into 96‐well plates at a density of 8 × 10^3^ cells/well. After overnight adherence, cells were infected with OV‐Q1, OV‐IL15C, or OV‐IL15C‐CBD at an MOI of 2, 10, or 50. Following 2 h of incubation, the viral inoculum was replaced with fresh medium containing 1% FBS, and cells were cultured for an additional 48 h. Cell viability was assessed using the Cell Counting Kit‐8 (CCK‐8, Yeasen). Briefly, 10 µL of CCK‐8 reagent was added to each well, and plates were incubated for 2 h at 37°C in the dark. Absorbance at 450 nm was measured using a microplate reader. Cytolytic activity was quantified based on relative absorbance values.

### 4.11 Quantification of E1A Genome Replication by qPCR

4T1, EMT6, SUM159, LLC, and A549 cells were seeded at 5 × 10^4^ cells/well in 24‐well plates and cultured overnight. The medium was replaced with RPMI‐1640 or DMEM containing 1% FBS, and cells were infected with OV‐Q1, OV‐IL15C, or OV‐IL15C‐CBD at an MOI of 10. After 2 h of adsorption, the inoculum was removed and replaced with fresh 1% FBS medium. At 48 h post‐infection, cells and supernatants were collected, and genomic DNA was extracted using a genomic DNA extraction kit (TIANGEN). Standard curves were generated from plasmids containing the target E1A fragment, and SYBR Green‐based qPCR was performed to calculate absolute E1A copy numbers using primers listed in Table S5.

### 4.12 Assessment of Immunogenic Cell Death

4T1 cells were seeded at 1 × 10^5^ cells/well in 12‐well plates and infected with the respective oncolytic adenoviruses at an MOI of 2, with an uninfected group serving as a control. At 48 h post‐infection, conditioned supernatants were collected and centrifuged at 1,000 × *g* for 5 min at 4°C to remove cellular debris. The clarified extracellular fractions were assayed using a Mouse HMGB1 ELISA Kit (Elabscience). Absorbance was measured at 450 nm, and HMGB1 concentrations were interpolated from a freshly prepared standard curve.

### 4.13 Quantification of IL‐15 Complex Secretion

4T1 and SUM159 cells were infected with OV‐Q1, OV‐IL15C, or OV‐IL15C‐CBD at an MOI of 10. After a 2 h incubation, the inoculum was removed and replaced with fresh complete medium. Cells were cultured for an additional 48 h, after which supernatants were harvested. The concentration of secreted IL‐15 and IL‐15Rα complexes was measured using a mouse IL‐15/IL‐15Rα complex ELISA kit (MultiSciences) in accordance with the manufacturer's instructions.

### 4.14 Co‐Culture of Engineered Oncolytic Adenoviruses with Splenocytes for Tumor Cell Killing

4T1‐luciferase cells were seeded in 96‐well plates at a density of 8 × 10^3^ cells/well. After adherence, the following experimental groups were established: (i) splenocytes alone; (ii) oncolytic adenovirus alone (OV‐Q1, OV‐IL15C, or OV‐IL15C‐CBD); (iii) oncolytic adenovirus combined with splenocytes (OV‐Q1 + splenocytes, OV‐IL15C + splenocytes, OV‐IL15C‐CBD + splenocytes). For splenocyte groups, red blood cell‐lysed splenocytes from BALB/c mice were added at a splenocyte‐to‐tumor cell ratio of 3:1. For oncolytic adenovirus treatment, the MOI was set at 2. Plates were incubated at 37°C with 5% CO_2_ for 48 h. After incubation, luciferase substrate was added, and luminescence was measured using a VISQUE in vivo imaging system (Viewworks). The reduction in tumor cell viability was quantified based on the mean luciferase luminescence intensity.

### 4.15 Splenocyte Activation Assay

Six‐well plates were pre‐coated overnight at 4°C with anti‐CD3 antibody (1 µg/mL; ThermoFisher), rinsed with PBS, and seeded with mouse splenocytes (1 × 10^6^ cells/well) after red blood cell lysis. Stimulation conditions included: recombinant IL15C or IL15C‐CBD (2 µg/mL) and CBD protein (2 µg/mL) as an additional control, supernatants from 4T1 cells infected with OV‐Q1, OV‐IL15C, or OV‐IL15C‐CBD (MOI = 2, 48 h; supplemented with FBS to a final concentration of 10%), and anti‐CD28 antibody (5 µg/mL; ThermoFisher). Dose‐dependent effects were evaluated by testing IL15C at 10 µg/mL in addition to the standard 2 µg/mL concentration under identical stimulation conditions. For NK cell‐specific assays, CD3/CD28 stimulation was omitted. Collagen co‐incubation experiments were designed to assess whether stromal collagen modulates IL15C‐CBD‐mediated lymphocyte activation. Six‐well plates were pre‐coated with type I collagen (10 µg/mL), type III collagen (20 µg/mL), or a combination of both (type I at 10 µg/mL plus type III at 20 µg/mL), with uncoated wells serving as controls. Splenocytes were then seeded and stimulated with either recombinant IL15C‐CBD protein (2 µg/mL) or supernatants from OV‐IL15C‐CBD‐infected 4T1 cells. All other stimulation and staining procedures were identical to the standard assay. Splenocytes were incubated for 48 h to assess activation and for 72 h to evaluate proliferation. Before flow cytometry, cells were stained with fluorophore‐conjugated antibodies against markers for T cells (CD3, CD4, CD8, IFN‐γ, GZMB, and Ki67) and NK cells (CD45, CD49b, IFN‐γ, GZMB, and Ki67). All antibodies were purchased from BioLegend and used at recommended dilutions. Cell viability was assessed using Zombie NIR viability dye (1:1000; BioLegend). Fc receptors were blocked with anti‐CD16/32 antibody (1:200; BioLegend) before staining. Samples were acquired on a flow cytometer and analyzed using FlowJo software (v10.9.0).

### 4.16 Tumor Model

Orthotopic TNBC and breast tumor models were established by injecting 4T1 or EMT6 cells (6 × 10^6^ cells in 50 µL PBS) into the second mammary fat pad on the right flank of female BALB/c mice. Tumor dimensions were recorded with electronic calipers, and volumes were calculated using the formula: V = L × W^2^/2. Mice were euthanized when tumor volumes exceeded 1,500 mm^3^ or upon signs of distress in accordance with ethical guidelines.

### 4.17 In Vivo Viral Biodistribution and Early Stromal Interaction

When 4T1 tumor volumes reached approximately 100 mm^3^, mice were randomly assigned to four groups and received a single intratumoral injection of PBS, OV‐Q1, OV‐IL15C, or OV‐IL15C‐CBD (1 × 10^7^ PFU for viral treatments). At 48 h post‐injection, a subset of tumors from the OV‐IL15C and OV‐IL15C‐CBD cohorts was excised and processed for multiplex immunofluorescence staining targeting type I collagen, type III collagen, adenovirus type 5 hexon, IL‐15, and nuclei to simultaneously visualize early viral distribution, transgene expression, and stromal collagen anchoring. In parallel, tumor tissues and major organs (liver, spleen, lung, and kidney) were harvested from all four groups for viral biodistribution analysis. Tissue samples (100 mg) were homogenized in 500 µL of ice‐cold PBS, centrifuged at 12,000 × *g* for 10 min at 4°C, and subjected to genomic DNA extraction. Absolute E1A copy numbers were quantified via SYBR Green‐based qPCR to assess early tissue‐specific viral genome replication.

### 4.18 In Vivo Immune Profiling

When tumor volumes reached approximately 100 mm^3^, mice were randomly assigned to four treatment groups: PBS, OV‐Q1, OV‐IL15C, and OV‐IL15C‐CBD. Each group received three intratumoral injections of PBS or the corresponding adenovirus (1 × 10^7^ PFU/mouse) on days 0, 2, and 4. Tumors were excised on day 7 post‐final injection, mechanically and enzymatically dissociated into single‐cell suspensions, and processed for flow cytometric analysis. Cells were stained with fluorochrome‐conjugated antibodies to profile tumor‐infiltrating lymphocytes and myeloid populations, including T cells (CD3, CD4, CD8, Foxp3, IFN‐γ, and GZMB), NK cells (CD45, CD49b, IFN‐γ, and GZMB), and TAMs (CD11b, F4/80, CD86, and CD206). All antibodies were purchased from BioLegend and used at recommended dilutions. Cell viability was assessed using Zombie NIR viability dye (1:1000; BioLegend). Fc receptors were blocked with anti‐CD16/32 antibody (1:200; BioLegend) before surface and intracellular staining. Flow cytometric acquisition was performed using a standard analyzer, and data were analyzed with FlowJo software (v10.9.0). In parallel, a subset of tumors excised on day 7 post‐final injection was fixed, sectioned, and subjected to CD8 immunofluorescence staining to spatially resolve the distribution of cytotoxic T lymphocytes within the tumor architecture.

### 4.19 Cytokine Quantification

On day 7 following the final treatment, tumor tissues and serum samples were harvested from each experimental group. Tumor specimens (∼120 mg) were homogenized in 600 µL of ice‐cold PBS, and the resulting lysates were centrifuged at 12, 000 ×  g for 10 min at 4°C to collect supernatants. Levels of IL‐6, IL‐10, IFN‐γ, and TNF‐α in both tumor lysates and serum were measured using ELISA kits (MultiSciences) according to the manufacturer's protocols.

### 4.20 RNA Sequencing Analysis

Tumor tissues were collected from each group on day 7 post‐final treatment. Total RNA was extracted using TRIzol reagent (Invitrogen) according to the manufacturer's protocol. RNA quality and integrity were assessed before library construction. RNA sequencing libraries were prepared and sequenced on an Illumina platform by GENEWIZ (Suzhou, China).

### 4.21 In Vivo Antitumor Efficacy and Safety Evaluation

BALB/c mice bearing orthotopic 4T1 or EMT6 tumors (grouped and treated as described above) were monitored every two days for tumor growth using electronic calipers. Mice were euthanized when tumor volumes exceeded 1500 mm^3^, and survival was recorded over a 30‐day observation period for both models. On day 7 following the final treatment, tumors were excised and weighed to assess therapeutic efficacy. Additionally, body weight changes were monitored throughout the study for both the 4T1 and EMT6 models to evaluate systemic tolerability. Excised 4T1 tumor tissues were partially fixed in paraffin, sectioned, and subjected to histological and immunofluorescence analyses, including H&E staining, TUNEL assay, and Ki67 immunostaining. Slide scanning and analysis were performed using the Olympus SLIDEVIEW VS200 research slide scanner. Systemic toxicity in the 4T1 model was evaluated by harvesting major organs (heart, liver, spleen, lung, and kidney) on day 7 post‐treatment for H&E staining. Serum samples from 4T1 tumor‐bearing mice were analyzed using a fully automated biochemical analyzer (Rayto, China) to evaluate hepatic and renal function by measuring biochemical indicators, including ALB, ALP, ALT, AST, TBIL, DBIL, TBA, UA, CREA, and γ‐GT.

### 4.22 Statistical Analysis

All the data are presented as the mean ± SD. Survival curves were analyzed using the log‐rank (Mantel‐Cox) test. Comparisons between two groups were conducted using an unpaired two‐tailed Student's t‐test or nonparametric test, whereas multiple group comparisons were assessed by one‐way or two‐way ANOVA followed by Tukey's multiple comparisons test. Statistical analyses were performed using GraphPad Prism version 10.0. A *p* value less than 0.05 was considered statistically significant. Non‐significant differences are indicated as ns.

## Author Contributions

Conceptualization: Y.Y., X.L., J.D., and X.G. Methodology: Y.Y., X.L., J.D., and J.X. Investigation: Y.Y., X.L., J.D., J.Z., H.Z., R.Y., S.L., and L.Z. Data curation: Y.Z. and J.W. Formal analysis: Y.Y., X.L., and J.D. Visualization: Y.Z. and R.S. Funding acquisition: X.G., X.L., and R.S. Supervision: X.G., C.Z., and H.L. Writing – original draft: Y.Y., X.L., and J.D. Writing – review & editing: Y.Y., X.L., J.D., H.L., C.Z., and X.G.

## Funding

This work was supported by the National Natural Science Foundation of China (82572390), the Shanghai Municipal Science and Technology Major Project (ZD2021CY001), the Postdoctoral Fellowship Program of China Postdoctoral Science Foundation (GZC20250997, GZC20252365), and the China Postdoctoral Science Foundation (2024M760539, 2025M773560).

## Ethics Approvals

The Animal Ethics Committee of Fudan University reviewed and approved all experimental protocols involving animals in this study (Approval No. 20250227‐179). All in vivo procedures strictly adhered to institutional guidelines and the Guide for the Care and Use of Laboratory Animals.

## Conflicts of Interest

The authors declare no conflicts of interest.

## Supporting information




**Supporting File**: advs77691‐sup‐0001‐SuppMat.docx.

## Data Availability

All data needed to evaluate the conclusions in the paper are present in the paper and/or the Supplementary Materials.
