# Supplementary material for: Collagen‐Binding IL‐15 Immunocytokine Armed Oncolytic Adenovirus Establishes a Self‐Reinforcing Immunostimulatory Milieu to Potentiate Antitumor Immunity
Source: Adv Sci (Weinh). 2026 Sep 16:e77691. Online ahead of print. doi: 10.1002/advs.77691 (PMC13580172; doi:10.1002/advs.77691)
Supplement: Supplementary file 1 [file ADVS-9999-e77691-s001.docx]

**Supporting Information**

**Collagen-Binding IL-15 Immunocytokine Armed Oncolytic Adenovirus Establishes a Self-Reinforcing Immunostimulatory Milieu to Potentiate Antitumor Immunity**

Yaqi Yang^1, †^, Xiaoxiao Liu^1, †^, Junqiang Ding^1, 2, †^, Jinliang Xu^3^, Jianhua Zhang^1^, Hanchang Zhang^1^, Rong Yang^1, 4^, Sha Li^1^, Lu Zheng^1^, Runping Su^1^, Yue Zhao^1^, Jianxiao Wu^1^, Hao Li^5, *^, Changyou Zhan^3, *^, Xihui Gao^1, *^

**Table of contents**

**Supplementary Figs**

Supplementary Fig. 1	1

Supplementary Fig. 2	2

Supplementary Fig. 3	3

Supplementary Fig. 4	4

Supplementary Fig. 5	5

Supplementary Fig. 6	5

Supplementary Fig. 7	5

Supplementary Fig. 8	6

Supplementary Fig. 9	6

Supplementary Fig. 10	7

Supplementary Fig. 11	8

Supplementary Fig. 12	9

Supplementary Fig. 13	10

Supplementary Fig. 14	11

Supplementary Fig. 15	12

Supplementary Fig. 16	14

Supplementary Fig. 17	15

Supplementary Fig. 18	16

Supplementary Fig. 19	16

Supplementary Fig. 20	17

Supplementary Fig. 21	17

Supplementary Fig. 22	18

Supplementary Fig. 23	18

Supplementary Fig. 24	19

Supplementary Fig. 25	19

Supplementary Fig. 26	20

Supplementary Fig. 27	20

Supplementary Fig. 28	21

Supplementary Fig. 29	22

Supplementary Fig. 30	23

Supplementary Fig. 31	24

Supplementary Fig. 32	25

Supplementary Fig. 33	26

Supplementary Fig. 34	27

Supplementary Fig. 35	28

Supplementary Fig. 36	29

Supplementary Fig. 37	30

Supplementary Fig. 38	31

Supplementary Fig. 39	32

Supplementary Fig. 40	32

**Supplementary Tables**

Supplementary Table 1	33

Supplementary Table 2	33

Supplementary Table 3	34

Supplementary Table 4	35

Supplementary Table 5	36

**Supplementary Figs**

**Supplementary Fig. 1**

**Supplementary Fig. 1 |** Relationship between IL-15 expression and OS (**a**) and DFS (**b**) was analyzed. Relationship between IL-15Rα expression and OS (**c**) and DFS (**d**) was evaluated.

**Supplementary Fig. 2**

**Supplementary Fig. 2 |** Pan-cancer correlation analysis between the expression of IL-15 (**a**) and IL-15Rα (**b**) and 24 types of immune cells using the ssGSEA algorithm.

**Supplementary Fig. 3**

**Supplementary Fig. 3 |** Molecular docking of IL15C with IL-15Rβ (**a**) and IL-15Rγc (**d**). The molecular docking of CBD-IL15C with IL-15Rβ (**b**) and IL-15Rγc (**e**). Molecular docking of IL15C-CBD with IL-15Rβ (**c**) and IL-15Rγc (**f**).

**Supplementary Fig. 4**

**Supplementary Fig.4 |** Cluster analysis and merged conformations of hydrophobic domains for IL15C (**a**) and IL15C-CBD (**b**). Representative 3D structures of IL15C (**c**) and IL15C-CBD (**d**) overlaid with intramolecular hydrogen bond networks.

**Supplementary Fig. 5**

**Supplementary Fig. 5 |** Western blot analysis of IL15C and IL15C-CBD.

**Supplementary Fig. 6**

**Supplementary Fig. 6 |** (**a**) Schematic representation of the gWIZ expression vector encoding the CBD. (**b**) SDS-PAGE analysis of purified CBD (lane M, protein marker; lane 1, CBD). (**c**) Dose-dependent binding of CBD to type I collagen evaluated by ELISA (n = 3/group). ELISA analysis of the binding capacities of CBD to IL-15Rβ (**d**) and IL-15Rγc (**e**) (n = 3/group). The data are presented as the mean ± SD. Statistical significance was assessed using one-way ANOVA with Tukey’s multiple comparisons (**c**-**e**). Image created with BioRender. com, with permission (**a**).

**Supplementary Fig. 7**

**Supplementary Fig. 7 |** SDS-PAGE analysis of purified IL-15Rβ with Fc tags. (lane M, protein marker; lane 1, IL-15Rβ-Fc).

**Supplementary Fig. 8**

**Supplementary Fig. 8 |** SDS-PAGE analysis of purified IL-15Rγc with Fc tags. (lane M, protein marker; lane 1, IL-15Rγc-Fc).

**Supplementary Fig. 9**

**Supplementary Fig. 9 |** ELISA assay evaluating the binding capacity of IL15C to type Ⅰ collagen at different concentrations (n=3/group). The Data are presented as the mean ± SD. Statistical significance was determined using one-way ANOVA with Tukey’s multiple comparisons. ns indicates no statistically significant difference.

**Supplementary Fig. 10**

**Supplementary Fig. 10 |** ELISA assay evaluating the binding capacity of IL15C to type Ⅲ collagen at different concentrations (n=3/group). The Data are presented as the mean ± SD. Statistical significance was determined using one-way ANOVA with Tukey’s multiple comparisons. ns indicates no statistically significant difference.

**Supplementary Fig. 11**

**Supplementary Fig. 11 |** Representative immunofluorescence staining of type I collagen (green), type III collagen (red), and nuclei (DAPI, blue) in BALB/c mouse tail (**a**), mouse skin (**b**), and 4T1 tumor tissue (**c**). n = 3mice/group; Scale bar, 1 mm.

**Supplementary Fig. 12**

**Supplementary Fig. 12 |** Representative *ex* *vivo* immunofluorescence images of 4T1 tumor cryosections incubated with IL15C or IL15C-CBD and stained for type I collagen (green), type III collagen (red), IL-15 (white), and nuclei (DAPI, blue). n = 3mice/group; Scale bar, 2 mm.

**Supplementary Fig. 13**

**Supplementary Fig. 13 | (a)** Gating strategy for flow cytometric identification of CD4^+^GZMB^+^ T cells, CD8^+^GZMB^+^ T cells, CD45^+^CD49b^+^IFN-γ^+^ cells, and CD45^+^CD49b^+^GZMB^+^ cells (related to Fig. 1r, s, u, v). Representative flow cytometry plots for CD4^+^GZMB^+^ T cells **(b)**, CD8^+^GZMB^+^ T cells **(c)**, CD45^+^CD49b^+^IFN-γ^+^ cells **(d)**, and CD45^+^CD49b^+^GZMB^+^ cells **(e)**, corresponding to quantifications in Fig. 1r, s, u, and v, respectively.

**Supplementary Fig. 14**

**Supplementary Fig. 14 | (a)** Gating strategy for flow cytometric identification of CD8^+^Ki67^+^ T cells and CD45^+^CD49b^+^Ki67^+^ cells (related to Fig. 1t, w). Representative flow cytometry plots for CD8^+^Ki67^+^ T cells **(b)** and CD45^+^CD49b^+^Ki67^+^ cells **(c)**, corresponding to quantifications in Fig. 1t and w, respectively.

**Supplementary Fig. 15**

**Supplementary Fig. 15 |** (**a**) Schematic of the experimental workflow. Splenocytes were isolated and coincubated with recombinant IL15C-CBD protein for 48 hours in the presence of the indicated collagen substrates, followed by immunophenotypic characterization by flow cytometry. Experimental cohorts comprised an uncoated control (G1), isolated type I (G2) or type III (G3) collagen coatings, and a combined type I and type III collagen matrix (G4). (**b**) Gating strategy for flow cytometric identification of CD8^+^IFN-γ^+^ T cells, CD8^+^GZMB^+^ T cells, CD45^+^CD49b^+^IFN-γ^+^ cells, and CD45^+^CD49b^+^GZMB^+^ cells. Representative flow cytometry plots (**c**) and quantification (**d**) of CD8^+^IFN-γ^+^ T cells. Representative flow cytometry plots (**e**) and quantification (**f**) of CD8^+^GZMB^+^T cells. Representative flow cytometry plots (**g**) and quantification (**h**) of CD45^+^CD49b^+^IFN-γ^+^ cells. Representative flow cytometry plots (**i**) and quantification (**j**) of CD45^+^CD49b^+^GZMB^+^ cells. n = 3/group; the data are presented as the mean ± SD. Statistical significance was assessed using one-way ANOVA with Tukey’s multiple comparisons (**d**, **f**, **h**, and **j**); ns indicates no significant difference. Image created with BioRender. com, with permission (**a**).

**Supplementary Fig. 16**

**Supplementary Fig. 16 |** (**a**) Gating strategy for flow cytometric identification of CD4^+^IFN-γ^+^ T cells, CD4^+^GZMB^+^ T cells, CD8^+^IFN-γ^+^ T cells, CD8^+^GZMB^+^ T cells, CD45^+^CD49b^+^IFN-γ^+^ cells, and CD45^+^CD49b^+^GZMB^+^ cells. Representative flow cytometry plots (**b**) and quantification (**c**) of CD4^+^IFN-γ^+^ T cells. Representative flow cytometry plots (**d**) and quantification (**e**) of CD4^+^GZMB^+^ T cells. Representative flow cytometry plots (**f**) and quantification (**g**) of CD8^+^IFN-γ^+^ T cells. Representative flow cytometry plots (**h**) and quantification (**i**) of CD8^+^GZMB^+^ T cells. Representative flow cytometry plots (**j**) and quantification (**k**) of CD45^+^CD49b^+^IFN-γ^+^ cells. Representative flow cytometry plots (**l**) and quantification (**m**) of CD45^+^CD49b^+^GZMB^+^ cells. n = 3/group; the data are presented as the mean ± SD. Statistical significance was assessed using t-tests and nonparametric tests (**c**, **e**, **g**, **i**, **k**, and **m**).

**Supplementary Fig. 17**

**Supplementary Fig. 17 |** (**a**) Gating strategy for flow cytometric identification of CD8⁺Ki67⁺ T cells and CD45⁺CD49b⁺Ki67⁺ cells. Representative flow cytometry plots (**b**) and quantification (**c**) of CD8⁺Ki67⁺ T cells. Representative flow cytometry plots (**d**) and quantification (**e**) of CD45⁺CD49b⁺Ki67⁺ cells. n = 3/group; the data are presented as the mean ± SD. Statistical significance was assessed using t-tests and nonparametric tests (**c**, **e**).

**Supplementary Fig. 18**

**Supplementary Fig. 18 |** (**a**) Schematic diagram of the construction and packaging of the OV-Q1 viral vector. (**b**) Schematic diagram of the construction and packaging of the OV-IL15C viral vector. Image created with BioRender. com, with permission (**a**, **b**).

**Supplementary Fig. 19**

**Supplementary Fig. 19 |** Absolute qPCR quantification of E1A gene copies in 4T1 (**a**), EMT6 (**b**), SUM159 (**c**), LLC (**d**), and A549 (**e**) cells under the indicated treatments (n = 4/group). Statistical significance was assessed using one-way ANOVA with Tukey’s multiple comparisons (**a-e**); ns indicates no significant difference.

**Supplementary Fig. 20**

**Supplementary Fig. 20 |** Dose-dependent oncolytic activity of OV-Q1, OV-IL15C, and OV-IL15C-CBD in LLC cells. Tumor cells were infected with the viruses at the indicated MOIs, and cell viability was assessed by a CCK-8 assay 48 hours post-infection (n = 6/group). The data are presented as the mean ± SD. Statistical significance was assessed using two-way ANOVA with Tukey’s multiple comparisons. ns indicates no significant difference.

**Supplementary Fig. 21**

**Supplementary Fig. 21 |** Dose-dependent oncolytic activity of OV-Q1, OV-IL15C, and OV-IL15C-CBD in A549 cells. Tumor cells were infected with the viruses at the indicated MOIs, and cell viability was assessed by a CCK-8 assay 48 hours post-infection (n = 6/group). The data are presented as the mean ± SD. Statistical significance was assessed using two-way ANOVA with Tukey’s multiple comparisons. ns indicates no significant difference.

**Supplementary Fig. 22**

**Supplementary Fig. 22 |** Extracellular HMGB1 concentrations under the indicated treatments (n = 4/group). The data are presented as the mean ± SD. Statistical significance was assessed using one-way ANOVA with Tukey’s multiple comparisons. ns indicates no significant difference.

**Supplementary Fig. 23**

**Supplementary Fig. 23 |** (**a**) Gating strategy used to identify CD8^+^IFN-γ^+^ T cell subsets by flow cytometry (related to Fig. 3g). (**b**) Representative flow cytometry plots corresponding to the quantification of CD8^+^IFN-γ^+^ T cells shown in Fig. 3g.

**Supplementary Fig. 24**

**Supplementary Fig. 24 |** (**a**) Gating strategy used to identify CD8^+^Ki67^+^ T cell subsets by flow cytometry (related to Fig. 3h). (**b**) Representative flow cytometry plots corresponding to the quantification of CD8^+^Ki67^+^ T cells shown in Fig. 3h.

**Supplementary Fig. 25**

**Supplementary Fig. 25 |** (**a**) Gating strategy used to identify CD4^+^GZMB^+^ T cell subsets by flow cytometry (related to Fig. 3i). (**b**) Representative flow cytometry plots corresponding to the quantification of CD4^+^GZMB^+^ T cells shown in Fig. 3i.

**Supplementary Fig. 26**

**Supplementary Fig. 26 |** (**a**) Gating strategy used to identify CD45^+^CD49b^+^GZMB^+^ cell subsets by flow cytometry (related to Fig. 3j). (**b**) Representative flow cytometry plots corresponding to the quantification of CD45^+^CD49b^+^GZMB^+^ cells shown in Fig. 3j.

**Supplementary Fig. 27**

**Supplementary Fig. 27 |** (**a**) Gating strategy used to identify CD45^+^CD49b^+^ IFN-γ^+^ cell subsets by flow cytometry (related to Fig. 3k). (**b**) Representative flow cytometry plots corresponding to the quantification of CD45^+^CD49b^+^ IFN-γ^+^ cells shown in Fig. 3k.

**Supplementary Fig. 28**

**Supplementary Fig. 28 |** (**a**) Gating strategy used to identify CD45^+^CD49b^+^ Ki67^+^ cell subsets by flow cytometry (related to Fig. 3l). (**b**) Representative flow cytometry plots corresponding to the quantification of CD45^+^CD49b^+^ Ki67^+^ cells shown in Fig. 3l.

**Supplementary Fig. 29**

**Supplementary Fig. 29 |** (**a**) Schematic of the experimental workflow. Supernatants were harvested from OV-IL15C-CBD-infected cells and coincubated with immune cells for 48 hours in the presence of designated collagen substrates. Experimental cohorts comprised an uncoated control (G1), isolated type I (G2) or type III (G3) collagen coatings, and a combined type I and type III collagen matrix (G4). (**b**) Gating strategy for flow cytometric identification of CD45^+^CD49b^+^GZMB^+^ cells. Representative flow cytometry plots (**c**) and quantification (**d**) of CD45^+^CD49b^+^GZMB^+^ cells. n = 3/group; the data are presented as the mean ± SD. Statistical significance was assessed using one-way ANOVA with Tukey’s multiple comparisons; ns indicates no significant difference. Image created with BioRender. com, with permission (**a**).

**Supplementary Fig. 30**

**Supplementary Fig. 30 | (a)** Gating strategy for flow cytometric identification of tumor-infiltrating CD8^+^, CD4^+^IFN-γ^+^, CD4^+^GZMB^+^, CD8^+^IFN-γ^+^, and CD8^+^GZMB^+^ cell subsets (related to Fig. 4b-g). Representative flow cytometry plots for tumor-infiltrating CD8^+^ T cells **(b)**, CD4^+^Foxp3^+^ Treg cells **(c)**, CD4^+^IFN-γ^+^ T cells **(d)**, CD4^+^GZMB^+^ T cells **(e)**, CD8^+^IFN-γ^+^ T cells **(f)**, and CD8^+^GZMB^+^ T cells **(g)**, corresponding to quantifications in Fig. 4b-g, respectively. Treatment groups included G1 (PBS), G2 (OV-Q1), G3 (OV-IL15C), and G4 (OV-IL15C-CBD).

**Supplementary Fig. 31**

**Supplementary Fig. 31 |** Representative whole-section immunofluorescence scans of 4T1 tumor cryosections from mice treated with intratumoral PBS, OV-Q1, OV-IL15C, or OV-IL15C-CBD, stained for CD8 (red) and nuclei (DAPI, blue). n = 3mice/group; Scale bar, 1 mm.

**Supplementary Fig. 32**

**Supplementary Fig. 32 | (a)** Gating strategy for flow cytometric identification of tumor-infiltrating CD45^+^CD49b^+^ cells, CD45^+^CD49b^+^IFN-γ^+^cells, and CD45^+^CD49b^+^GZMB^+^ cells (related to Fig. 4h-j). Representative flow cytometry plots for tumor-infiltrating CD45^+^CD49b^+^ cells **(b)**, CD45^+^CD49b^+^IFN-γ^+^ cells **(c)**, and CD45^+^CD49b^+^GZMB^+^ cells **(d)**, corresponding to quantifications in Fig. 4h-j, respectively. Treatment groups included G1 (PBS), G2 (OV-Q1), G3 (OV-IL15C), and G4 (OV-IL15C-CBD).

**Supplementary Fig. 33**

**Supplementary Fig. 33 | (a)** Gating strategy for flow cytometric identification of tumor-infiltrating CD11b^+^F4/80^+^CD86^+^ (M1-like) and CD11b^+^F4/80^+^CD206^+^ (M2-like) macrophage subsets (related to Fig. 4k, l). Representative flow cytometry plots for CD11b^+^F4/80^+^CD86^+^ macrophages **(b)** and CD11b^+^F4/80^+^CD206^+^ macrophages **(c)**, corresponding to quantifications in Fig. 4k and 4l, respectively. Treatment groups included G1 (PBS), G2 (OV-Q1), G3 (OV-IL15C), and G4 (OV-IL15C-CBD).

**Supplementary Fig. 34**

**Supplementary Fig. 34 |** Representative multiplex immunofluorescence images of 4T1 tumor cryosections collected 48 hours after intratumoral administration of OV-IL15C or OV-IL15C-CBD, stained for type I collagen (cyan), type III collagen (white), adenovirus type 5 hexon (red), IL-15 (yellow), and nuclei (DAPI, blue). **(a)** Whole-section scanning images. Scale bar, 1 mm. **(b)** High-magnification views of representative stromal regions. n = 3mice/group; Scale bar, 20 μm.

**Supplementary Fig. 35**

**Supplementary Fig. 35 |** (**a**) Experimental design for evaluating *in vivo* therapeutic efficacy. (**b**) Representative immunofluorescence staining of type I collagen (green), type III collagen (red), and nuclei (DAPI, blue) in EMT6 tumor tissue. n = 3mice/group; Scale bar, 1 mm. (**c**) Representative images of tumors excised on day 7 after the final treatment. Scale bar, 1 cm. (**d**) Changes in tumor weight in response to different treatments (n=5 mice/group). (**e**) Tumor inhibition rates for each treatment group (n=5 mice/group). Mean (**f**) and individual (**g**) tumor growth curves for each treatment group (n=7 mice/group). Treatment groups included G1 (PBS), G2 (OV-Q1), G3 (OV-IL15C), and G4 (OV-IL15C-CBD). The Data are presented as the mean ± SD. Statistical significance was assessed using one-way ANOVA with Tukey’s multiple comparisons (**d**, **e**); ns indicates no significant difference. Image created with BioRender. com, with permission (**a**).

**Supplementary Fig. 36**

**Supplementary Fig. 36 |** Kaplan-Meier survival curves of EMT6 tumor-bearing mice subjected to different treatments (n=7 mice/group). Statistical significance was assessed using the log-rank (Mantel-Cox) test. Treatment groups included G1 (PBS), G2 (OV-Q1), G3 (OV-IL15C), and G4 (OV-IL15C-CBD).

**Supplementary Fig. 37**

**Supplementary Fig. 37** **|** Body weight monitoring during treatment (n = 7 mice/group). Treatment groups included G1 (PBS), G2 (OV-Q1), G3 (OV-IL15C), and G4 (OV-IL15C-CBD).

**Supplementary Fig. 38**

**Supplementary Fig. 38 |** Representative H&E-stained images of the heart, liver, spleen, lung, and kidney from each treatment group (n=3 mice/group; scale bar, 100 μm). Treatment groups included G1 (PBS), G2 (OV-Q1), G3 (OV-IL15C), and G4 (OV-IL15C-CBD).

**Supplementary Fig. 39**

**Supplementary Fig. 39 |** Serum levels of ALB (**a**), ALP (**b**), ALT (**c**), AST (**d**), TBIL (**e**), DBIL (**f**), TBA (**g**), UA (**h**), CREA (**i**), and γ-GT (**j**) were measured across different treatment groups. Treatment groups included G1 (PBS), G2 (OV-Q1), G3 (OV-IL15C), and G4 (OV-IL15C-CBD).

**Supplementary Fig. 40**

**Supplementary Fig. 40 |** E1A copy numbers in tumor (**a**), liver (**b**), spleen (**c**), lung (**d**), and kidney (**e**), n = 3 mice/group. Data are presented as the mean ± SD. Statistical significance was assessed using one-way ANOVA with Tukey’s multiple comparisons (**a-e**); ns indicates no significant difference. Treatment groups included G1 (PBS), G2 (OV-Q1), G3 (OV-IL15C), and G4 (OV-IL15C-CBD).

**Supplementary Table 1**

**Quantitative parameters of hydrophobic clusters identified in IL15C**

| Cluster ID | Area | Number of contacts | Contacts/Residue | Area/Residue |
| --- | --- | --- | --- | --- |
| 0 | 3008.84 | 76 | 2.92 | 39.59 |
| 1 | 68.93 | 2 | 1 | 34.46 |
| 2 | 169.32 | 4 | 1.33 | 42.33 |
| 3 | 73.8 | 2 | 1 | 36.9 |

**Supplementary Table 2**

**Quantitative parameters of hydrophobic clusters identified in IL15C-CBD**

| Cluster ID | Area | Number of contacts | Contacts/Residue | Area/Residue |
| --- | --- | --- | --- | --- |
| 0 | 3218.28 | 79 | 3.04 | 40.74 |
| 1 | 105.05 | 2 | 1 | 52.53 |
| 2 | 167.77 | 4 | 1.33 | 41.94 |
| 3 | 110.59 | 2 | 1 | 55.3 |
| 4 | 3444.35 | 82 | 3.57 | 42 |
| 5 | 2907.78 | 60 | 3.33 | 48.46 |
| 6 | 56.52 | 2 | 1 | 28.26 |

**Supplementary Table 3**

**Intramolecular hydrogen bond parameters of IL15C**

| Network ID | Donor-Aceptor | Distance | Angle DHA (degrees) |
| --- | --- | --- | --- |
| 0 | ASN66-ND2 -- ASP9-OD2 | 2.31 | 120.75 |
| 1 | LYS95-NZ -- GLU93-OE1 | 2.9 | 160.46 |
| 2 | GLN109-NE2 -- ASN113-ND2 | 2.57 | 124.38 |
| 3 | ARG156-NH1 -- GLU54-OE1 | 3.2 | 160.38 |
| 3 | ARG156-NH1 -- GLU54-OE2 | 3.16 | 132.31 |
| 3 | ARG156-NH2 -- ASP23-OD1 | 2.93 | 144.74 |
| 3 | ARG156-NH2 -- THR24-OG1 | 3.19 | 134.65 |
| 3 | ARG156-NH2 -- GLU54-OE2 | 2.91 | 141.4 |
| 4 | ARG165-NH2 -- GLU47-OE2 | 3.01 | 168.13 |
| 3 | THR24-OG1 -- GLU54-OE2 | 2.93 | 134.94 |
| 5 | THR25-OG1 -- GLU94-OE2 | 2.87 | 162.01 |
| 6 | THR39-OG1 -- GLU83-OE2 | 2.51 | 158.44 |
| 7 | THR96-OG1 -- GLU99-OE2 | 2.95 | 174.91 |

**Supplementary Table 4**

**Intramolecular hydrogen bond parameters of IL15C-CBD**

| Network ID | Donor-Aceptor | Distance | Angle DHA (degrees) |
| --- | --- | --- | --- |
| 0 | ARG156-NH2 -- GLU54-OE2 | 3.28 | 170.37 |
| 1 | ASN160-ND2 -- ASP143-OD1 | 3.41 | 159.38 |
| 2 | GLN270-NE2 -- ASP278-OD2 | 2.58 | 163.14 |
| 3 | ARG325-NE -- ASP227-OD1 | 2.56 | 125.99 |
| 3 | ARG325-NE -- ASP227-OD2 | 3.47 | 174.53 |
| 3 | ARG325-NH2 -- ASP227-OD1 | 2.27 | 136.41 |
| 4 | ARG373-NE -- ASP381-OD1 | 3.33 | 153.28 |
| 0 | THR24-OG1 -- GLU54-OE2 | 2.67 | 166.37 |
| 5 | THR25-OG1 -- GLU94-OE2 | 2.98 | 169.79 |
| 6 | THR39-OG1 -- GLU83-OE2 | 2.65 | 164.86 |
| 7 | THR96-OG1 -- GLU99-OE2 | 3.12 | 173.22 |
| 8 | SER120-OG -- GLN18-OE1 | 2.22 | 146.37 |
| 9 | SER150-OG -- ASN148-OD1 | 2.73 | 136.16 |
| 0 | SER170-OG -- GLU54-OE1 | 3 | 173.39 |
| 10 | SER223-OG -- ASP221-OD1 | 2.69 | 124.18 |
| 10 | SER223-OG -- ASP221-OD2 | 2.79 | 160.04 |
| 11 | SER235-OG -- ASP233-OD1 | 2.77 | 151.63 |
| 12 | SER237-OG -- ASP338-OD2 | 2.93 | 161 |
| 2 | THR276-OG1 -- ASP278-OD2 | 3.25 | 152.78 |

**Supplementary Table 5**

**Primer details**

| **Gene** | **Forward (5′-3′)** | **Reverse (5′-3′)** |
| --- | --- | --- |
| E1A | CTTACTCACTTTTCCGCC | CTCTTCATCCTCGTCGTC |
